# Hepatic gene body hypermethylation is a shared epigenetic signature of murine longevity

**DOI:** 10.1371/journal.pgen.1007766

**Published:** 2018-11-21

**Authors:** Oliver Hahn, Thomas M. Stubbs, Wolf Reik, Sebastian Grönke, Andreas Beyer, Linda Partridge

**Affiliations:** 1 Max Planck Institute for Biology of Ageing, Cologne, Germany; 2 Cellular Networks and Systems Biology, CECAD, University of Cologne, Cologne, Germany; 3 Epigenetics Programme, The Babraham Institute, Cambridge, United Kingdom; 4 The Wellcome Trust Sanger Institute, Cambridge, United Kingdom; 5 Center for Molecular Medicine Cologne, University of Cologne, Cologne, Germany; 6 Department of Genetics, Evolution and Environment, Institute of Healthy Ageing, University College London, London, United Kingdom; Stanford University School of Medicine, UNITED STATES

## Abstract

Dietary, pharmacological and genetic interventions can extend health- and lifespan in diverse mammalian species. DNA methylation has been implicated in mediating the beneficial effects of these interventions; methylation patterns deteriorate during ageing, and this is prevented by lifespan-extending interventions. However, whether these interventions also actively shape the epigenome, and whether such epigenetic reprogramming contributes to improved health at old age, remains underexplored. We analysed published, whole-genome, BS-seq data sets from mouse liver to explore DNA methylation patterns in aged mice in response to three lifespan-extending interventions: dietary restriction (DR), reduced TOR signaling (rapamycin), and reduced growth (Ames dwarf mice). Dwarf mice show enhanced DNA hypermethylation in the body of key genes in lipid biosynthesis, cell proliferation and somatotropic signaling, which strongly correlates with the pattern of transcriptional repression. Remarkably, DR causes a similar hypermethylation in lipid biosynthesis genes, while rapamycin treatment increases methylation signatures in genes coding for growth factor and growth hormone receptors. Shared changes of DNA methylation were restricted to hypermethylated regions, and they were not merely a consequence of slowed ageing, thus suggesting an active mechanism driving their formation. By comparing the overlap in ageing-independent hypermethylated patterns between all three interventions, we identified four regions, which, independent of genetic background or gender, may serve as novel biomarkers for longevity-extending interventions. In summary, we identified gene body hypermethylation as a novel and partly conserved signature of lifespan-extending interventions in mouse, highlighting epigenetic reprogramming as a possible intervention to improve health at old age.

## Author summary

Dietary, genetic and pharmacological interventions can extend lifespan in diverse organisms and represent good models for developing therapies against age-related diseases in humans. However, whether these longevity interventions act by slowing down the effects of ageing, or inducing novel phenotypes to cope better with age-related stress, remains unclear. The epigenome is influenced by many factors including diet, drugs and genetic mutations, making it a possible candidate for linking longevity interventions. Here, we conducted a meta-analysis of published mouse liver datasets to profile changes in DNA methylation in response to three lifespan-extending interventions: reduced food intake, the drug rapamycin and a genetic mutation causing dwarfism. In dwarf mice, there was enhanced DNA methylation over specific genes involved in fatty metabolism and growth factor signaling. Reduced food intake and rapamycin treatment shared DNA methylation patterns with dwarf mice, revealing similar influences on the epigenome. Hypermethylated genes were also less active, which may protect dwarf and DR mice against liver steatosis and cancer. In addition, we developed novel DNA methylation biomarkers that could potentially predict longevity-extending interventions.

## Introduction

Dietary, genetic and pharmacological interventions can extend health- and lifespan in diverse model organisms whilst delaying the onset of age-related pathologies [1–3]. Understanding the underlying mechanisms holds the prospect of harnessing these benefits for prevention of age-related diseases in humans [3]. Dietary restriction (DR), in which food intake is reduced while avoiding malnutrition, is currently the most effective intervention to increase lifespan and health during ageing. DR extends lifespan in rodents and [2,4,5] improves health in primates [6–8] whilst protecting against age-related diseases such as cancer and diabetes [1,9,10]. In mice and rats, DR improves health- and lifespan also when applied in adult animals [11,12], demonstrating that the effects of DR are not result of altered development. Although the exact mediators remain elusive [13,14], studies in animal models have identified crucial roles of the regulatory, nutrient-sensing insulin/insulin-like growth factor/mTOR network [15–17] for improved health by DR. DR is further implicated to act through the somatotropic axis, an endocrine network regulating body growth via secretion of growth hormone (GH) from the pituitary gland and the downstream release of insulin-like growth factor (IGF-1) from the liver [15,18–21]. The specific molecular and physiological consequences of DR are, however, dependent on experimental variables including genetic background [10,22], gender [10,23] and food composition [2,7,24].

Consistent with its central role in DR-mediated longevity, genetically reduced somatotropic signaling can extend lifespan in mice [25–27]. For instance, Ames dwarf mice carry a homozygous mutation at the Prop1 locus, resulting in reduced GH secretion from the pituitary gland, with consequently reduced levels of circulating IGF-1 [19,28]. In addition to decreased body size, Ames dwarf mice exhibit high insulin sensitivity, increased stress resistance and delayed onset of neoplasia, which is specifically caused by GH deficiency [9,28–31]. Reduced somatotropic signaling also improves health- and lifespan in adult mice, as shown by inducible knockout of the Growth hormone receptor (GHR) [32] or antibody-mediated reduction of the IGF-1 receptor [33]. Lowered GH signaling is further associated with reduced activity of the mTOR network [34–37], which also has a well-established role in ageing [1,3]. Indeed, pharmacological inhibition of mTOR signaling via rapamycin increases lifespan in model organisms including adult mice [3,38].

DNA methylation is an epigenetic modification with a role in regulating gene transcription [39,40]. Methylation of CpG dinucleotides is prevalent in mammalian genomes, and is regarded as a relatively stable mark that is heritable across somatic cell divisions [41]. During ageing, however, the DNA methylation code slowly deteriorates, which may contribute to erosion of transcriptional control and to age-related pathologies such as cancer [39,42–44]. High-throughput analysis of human DNA methylation microarray data demonstrated that chronological and biological age are strongly correlated with methylation states of a few hundred CpGs, known as the epigenetic clock [42,45].

Epigenetic clocks have recently been developed for mice, and they are remarkably decelerated by DR, rapamycin treatment and reduced growth factor signaling [46–48]. In agreement with these observations, whole genome bisulfite sequencing (WGBS-seq) studies in DR [49,50], rapamycin-treated and Ames dwarf mice [50] demonstrated diminished age-related methylation changes across the mouse genome, maintaining a youthful profile over enhancers, promoters and heterochromatic regions [49,50]. Protection against age-related methylation changes and slowing of epigenetic clocks have therefore been proposed to contribute to healthy ageing. However, age-related changes in methylation pattern are largely uncorrelated with age-related changes in RNA transcript profiles [49,50], and it is thus unclear whether prevention of methylation changes has any functional consequences. In addition to slowing normal age-related changes, DR also causes hypermethylation in distinct regions of the genome. These DR-specific changes occurred predominantly in gene bodies, associated robustly with transcriptional repression, and the affected genes are enriched for key members of the lipogenic network controlled by Srebf1. Their methylation is paralleled by a healthier lipid profile, marked by decreased hepatic triacylglyceride accumulation and less elongated triglyceride-associated fatty acids [49].

In order to determine if changes in DNA methylation of genes in the lipogenic network are a general feature of DR, we conducted a meta-analysis of published WGBS-seq data for liver derived from two different strains of mice subjected to DR. We found strongly conserved hypermethylation of lipogenic Srebf1-target genes such as *Elovl6*, *Scd1* and *Fasn*, independent of genetic background, thereby revealing a robust epigenetic signature of DR. We also re-analyzed WGBS-seq profiles of aged rapamycin-treated and Ames dwarf mice, and found that DNA methylation is specifically remodeled in these interventions as well. Hyper- but not hypo-methylated regions of the genome coincide among different interventions, and are predominantly located in gene bodies. These patterns are induced at young age and do not result from delayed ageing alone, which points to an active mechanism driving their formation. Furthermore, joint methylome profiling identifies a limited set of common hypermethylated regions that could serve as a new DNA methylation-based biomarker for intervention outcome, independent of genetic background and gender.

Functionally, Ames dwarf mice shared hypermethylation patterns with rapamycin-treated mice over genes coding for signaling mediators of growth factors and growth hormone, whilst also showing hypermethylation over the same lipogenic Srebf1-target genes as DR-fed mice. In Ames dwarf mice specifically, we found extensive epigenetic silencing of the somatoropic signaling axis, including hypermethylation of *IGF-1* and oncogenic downstream targets *Egfr* and *Onecut1*. Analysis of published RNA-seq data of DR and Ames dwarf mice further revealed that hypermethylation patterns of gene bodies and RNA transcript profiles were strongly associated, pointing to a potentially causative role of DNA methylation in changes in gene expression in geroprotective interventions.

We thus found targeted epigenetic reprogramming to be a novel and partly conserved feature of DR, reduced TOR and reduced somatotropic signaling, and characterized how this new epigenotype could causally improve health in old animals.

## Results

### Epigenetic remodeling of lipid metabolism in response to dietary restriction is conserved across mouse strains

Lifespan-extending interventions such as DR shape the DNA methylome by delaying age-related methylation changes across the genome [49–52]. Long-term DR has further been shown to alter DNA methylation specifically over lipid metabolism genes in C3B6F1 hybrid mice. This epigenetic remodeling was induced already in young DR animals, becoming more marked in older animals and differed from the pattern of age-related methylation changes [49]. As these DR-related DNA methylation changes have so far only been described for C3B6F1 hybrid mice, we asked whether they are a general feature of DR, independent of genetic background.

**Fig 1:**
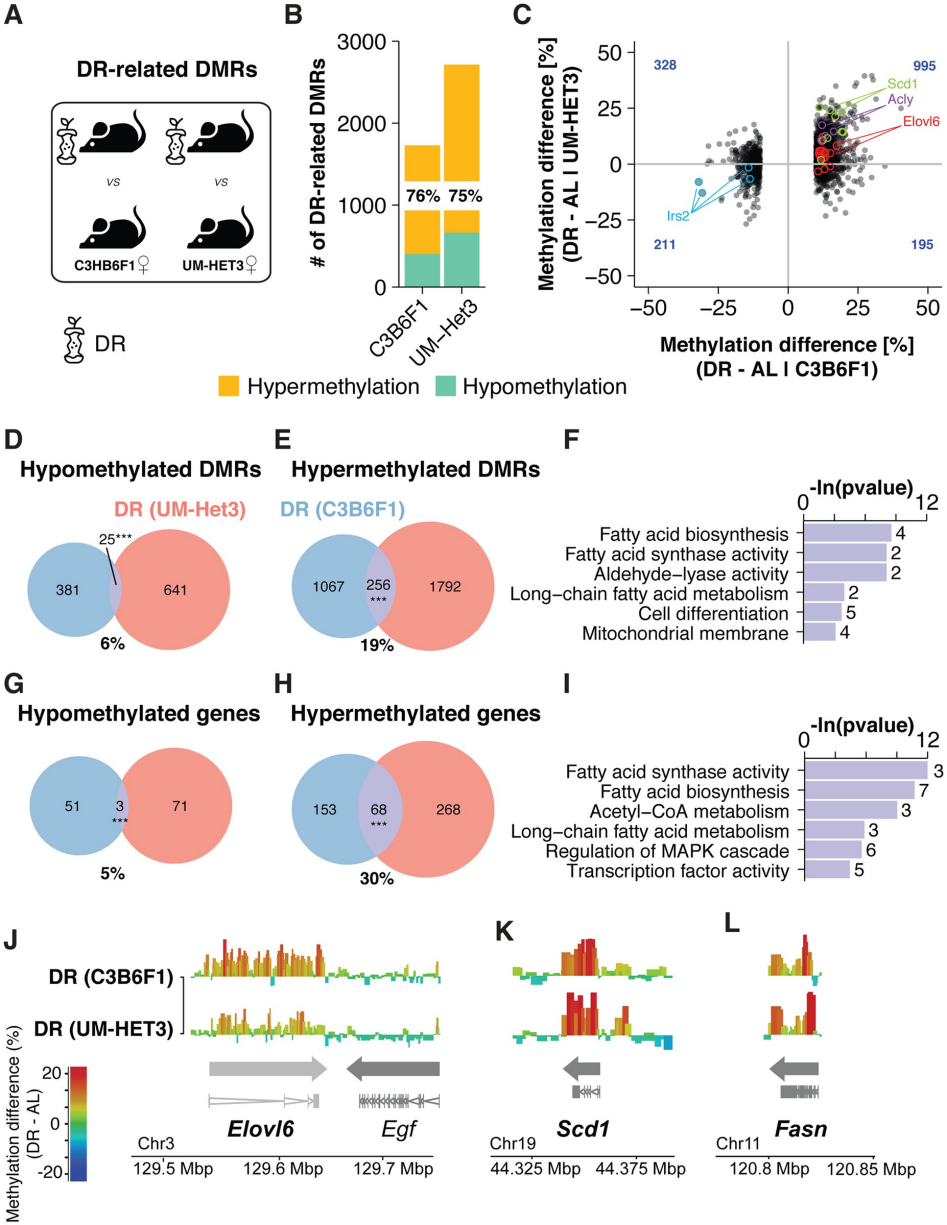
Dietary restriction-related methylation changes are conserved across mouse strains. (A) Schematic representation of the data sets analysed and comparisons made. We probed for Dietary Restricted (DR)-related DMRs at old age in B6C3F1 and UM-HET3♀ mice to test their conservation across mouse strains. Pre-processing was conducted in parallel for all experimental groups (B) Number of DMRs (p<0.05, ±10%< DNA methylation difference) between each DR cohort and its respective control. Proportion of hypermethylated DMRs are indicated in %. (C) Scatterplot comparison of bin-wise differences between DR-related DMRs in C3B6F1♀ mice versus changes in the same bin in dietary restricted UM-HET3♀. Bins overlapping the genes *Elovl6*, *Acly*, *Scd1* and *Irs2* are highlighted. Methylation differences in dietary restricted C3B6F1♀ mice were significantly associated with differences in dietary restricted UM-HET3♀ mice (One-sided *Fisher’s exact* test p <0.001). Number of bins in each quadrant is indicated in blue. (D, E) Venn diagrams depicting the overlap of significantly hypo- (D) and hypermethylated (E) DMRs in both DR cohorts. Proportions of overlap relative to the DR (C3B6F1♀) set are indicated in % (p-values; *** p<0.001, ** p<0.01, * p<0.05, *Fisher’s exact* test). (F) Functional enrichment of differentially methylated genes (with ≥ 2 common DMRs overlapping) detected in both DR cohorts (n = 51) (G, H) Venn diagrams depicting the overlap of significantly hypo- (G) and hypermethylated (H) genes (≥ 2 DMRs per cohort overlapping) present in both DR cohorts. Proportions of overlap relative to the DR (C3B6F1♀) set are indicated in % (p-values; *** p<0.001, ** p<0.01, * p<0.05, *Fisher’s exact* test). (I) Functional enrichment of differentially methylated genes (≥ 2 DMRs per cohort overlapping) detected in both DR cohorts (n = 71) (J-L) Differential methylation landscape of the *Elovl6*, *Egf* (J), *Scd1* (K) and *Fasn* (L) gene loci in both DR cohorts. The methylation increase upstream of *Scd1* did no pass statistical testing. Bins are represented as bars with color scale and height indicating methylation differences. Arrows indicate gene orientation; merged mRNA structure is depicted below.

We therefore compared two recently published datasets comprising whole-genome BS-seq data of liver samples (S1 Table); one from DR fed female F1 hybrid mice (C3B6F1♀) and the other from genetically heterogeneous DR fed female UM-HET3 mice (UM-HET3♀), and their *ad libitum* (AL) fed controls [49,50]. UM-HET3 mice are employed by the National Institute of Aging intervention testing program, thus representing a robust context to investigate longevity-related phenotypes [53]. DR increases the median lifespan of C3B6F1♀ from 902 to 1087 days (~ +25%) [49] and the mean lifespan of UM-HET3♀ mice from app. 836 to 1179 days (~ +39%; values for median lifespan were unavailable) [54]. The two datasets were generated from cohorts housed at different locations, undergoing DR from early adulthood on (DR starting at 3 and 2 months, respectively). Mice from the two studies received food of differing composition and were sacrificed at slightly different late ages (26 and 22 months, respectively) [49,50]. Furthermore, sample extraction and sequencing were conducted at different facilities and with differing sequencing conditions. Hence, a mixture of biological, experimental and technical confounding factors differed between the two DR datasets. Given the relatively low hepatic levels of non-CpG methylation, we centered our analysis on CpG methylation only. The two datasets were pre-processed and analyzed in parallel, using an unbiased and conservative quantification approach as described in [49] (for details see “Methods”). In brief, we binned the methylome using flexible-size windows covering 50 CpGs each, and overlapping adjacent windows by 25 CpGs. Focusing on methylation profiles of both DR fed C3B6F1♀ and UM-HET3♀ mice together with their respective controls, the total of four treatment groups (Fig 1A) yielded a set of 931,702 bins with a median bin size of 4 Kbp, covering 23 million CpGs in total. For each bin we calculated a single methylation value averaged across CpGs, ranging from 0 to 100% (unmethylated to fully methylated).

In both strains, DR significantly altered methylation levels of specific bins in comparison to corresponding age-matched controls (adjusted *p* < 0.05, Chi-squared test; minimal required difference cutoff of 10%), thus overcoming the need for further SNP allele frequency correction. In total, DR induced 1,729 differentially methylated regions (DMRs) in C3B6F1♀ and 2,714 DMRs in UM-HET3♀ mice (Fig 1B; S2 Table). About 75% of DR-related DMRs were hypermethylated, indicating a similar net effect of DR in each strain. Approximately 16% of DR-related DMRs detected in C3B6F1♀ mice showed a significant overlap with DR-related DMRs in UM-HET3♀ (*p* < 0.001, one-sided Fisher’s exact test), thus confirming a similar epigenetic remodeling independent of genetic background or experimental conditions (Fig 1C–1E). Interestingly, the overlap between hypermethylated DMRs (19%) was clearly larger than between hypomethylated DMRs (6%), suggesting that DR robustly alters the epigenome by locally increasing DNA methylation (Fig 1D and 1E). These common DR-related DMRs affected particularly key genes involved in lipid metabolism (Fig 1C and 1F; S3 Table).

The overlap between strains further increased (30% overlap) when comparing genes instead of individual DMRs (for details see “Methods”), suggesting differential methylation of entire gene loci as a conserved feature of DR (Fig 1G–1I). Indeed, DR induced similar differential methylation profiles over fatty acid synthesis genes in both genetic backgrounds, including *Elovl6*, *Scd1* and *Fasn* [55] (S4 Table), stretching over both introns and exons but not the promoter region or neighboring genes (Fig 1J–1L). Thus, targeted epigenetic reprogramming of lipogenic genes in the liver is a robust feature of DR independent of genetic background.

### Epigenetic remodeling under dietary restriction is tissue-specific

In order to assess if DR would remodel the methylome similarly in other tissues, we profiled previously published reduced-representation bisulfite-seq (RRBS-seq) data from blood of DR fed male F1 hybrid mice (B6D2F1♂) and DR fed male inbred mice (C57BL/6♂) [48] (S1A Fig). B6D2F1 mice were generated by crossing C57BL/6 with DBA/2J animals [48], thus have considerable genetic similarity with C57BL/6 mice. RRBS-seq allows differential analysis of individual CpGs due to higher read coverage per cytosine and lessened filtering by multiple testing correction [56,57]. In contrast to the liver, the majority of differentially methylated CpGs (dCpGs) showed significant hypomethylation in response to DR (S1B Fig). The effects of DR on DNA methylation in blood were well-conserved across strains, with 19 (hypermethylation) to 40% (hypomethylation) common dCpGs in both strains (S1C Fig), which is in line with previous studies [58]. We finally compared differentially methylated genes (for details, see “Methods”) in both tissues and found substantial conservation across strains but no consistent overlap across tissues (S1D and S1E Fig). Even though the discrepancy between blood and liver may be in part explained by the different BS-seq techniques, our analysis indicates that DR induces robust and highly tissue-specific epigenetic programs.

### Dietary restriction, reduced growth factor signaling and rapamycin treatment induced overlapping patterns of DNA methylation

**Fig 2:**
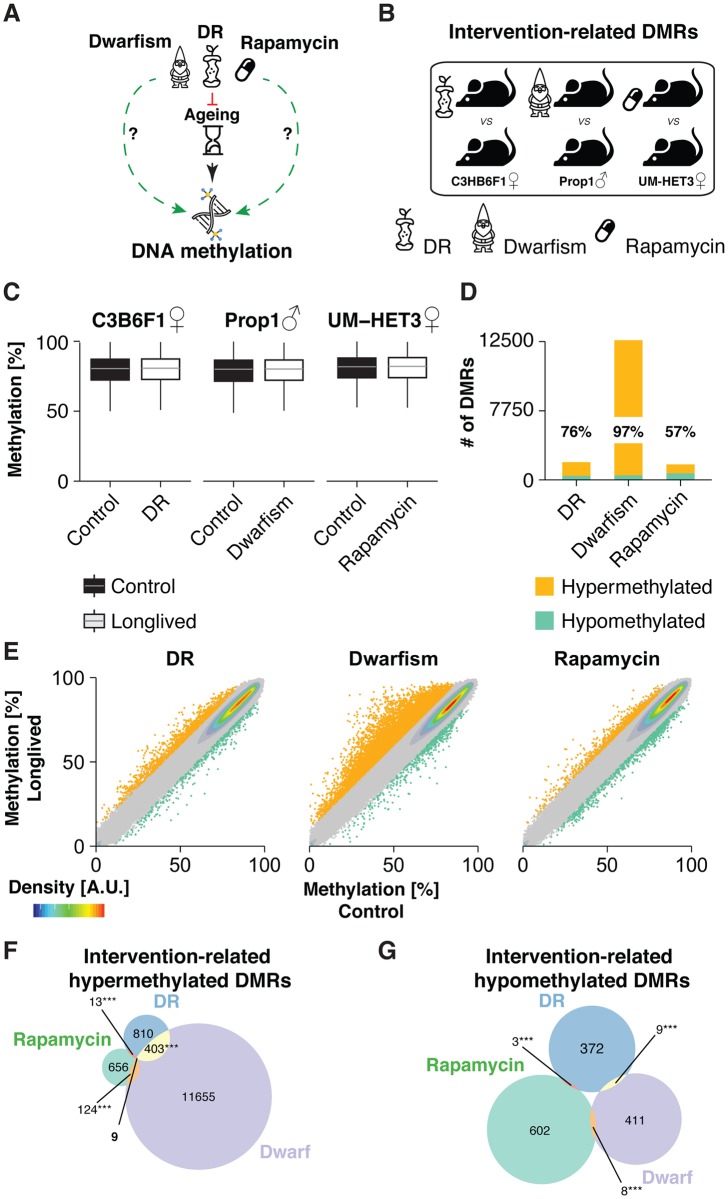
Cross-strain hepatic DNA methylation changes in response to longevity interventions. (A) Schematic of treatment comparisons: Various longevity interventions act on DNA methylation through retardation of ageing. We explored direct longevity-related (green arrow) effects on DNA methylation. (B) Schematic representation of the data sets analyzed and comparisons made. We probed for intervention-related DMRs at old age by comparing long-lived mice to their respective controls. Pre-processing was conducted for all shown treatment groups (C) Boxplot representation of global DNA methylation levels in the liver of aged DR, Ames Dwarf and Rapamycin treated mice next to their respective controls. Strain and sex are indicated above. (D) Number of significantly differentially methylated regions (DMRs) (p<0.05, ±10%< DNA methylation difference) between each longevity intervention and it’s respective control. Proportion of hypermethylated DMRs are indicated in %. (E) Scatterplot representation of methylation values of each bin in control and long-lived animals. Intervention-related DNA methylation changes are highlighted. Bins that were not differentially regulated (background) are represented in grey. Data density is indicated by color code. (F, G) Venn diagram depicting the overlap of significantly hyper- (F) and hypomethylated (G) DMRs under DR, Dwarfism and rapamycin treatment relative to the control groups at old age (p-values; *** p<0.001, ** p<0.01, * p<0.05, *Fisher’s exact* test).

Given the robust conservation of DR-related epigenetic reprogramming, we asked whether other longevity interventions also remodelled DNA methylation specifically (Fig 2A). We profiled the hepatic methylomes of female DR C3B6F1♀ mice, male Ames dwarf mice from a closed colony (strain referred to as Prop1♂) and rapamycin treated UM-HET3♀ mice, all relative to age-matched controls (Fig 2B) [49,50]. Compared to their control group, Ames dwarf mice show an almost 50% increase in mean lifespan (controls: 723 days; Ames dwarfs: 1076 days; values for median lifespan were unavailable) [25], while the median lifespan of UM-HET3♀ mice increases by ~26% in response to daily doses of 42 ppm rapamycin (controls: 896 days; rapamycin-treated: 1132 days) [59]. For DR, we included only C3B6F1♀ mice, given that WGBS-seq and RNA-seq data for both young and old mice were available only for this strain, and were required for downstream analyses (see below). With six treatment groups, our binning approach yielded a set of 922,788 bins covering 23 million CpGs with a median bin size of around 4 Kbp. Genome-wide levels of DNA methylation were largely unaffected by intervention or strain, with most bins showing levels in the range of 70–90% (Fig 2C).

In total, 15,075 DMRs showed significant methylation change in at least one intervention (intervention-related DMRs), which represents less than 1.5% of the entire methylome. Of these, DR induced 1,619 DMRs, rapamycin 1,415 and genetic dwarfism 12,619 (S5 Table). Both non-genetic interventions thus had a weaker impact on the epigenome than genetically reduced somatotropic signaling (Fig 2D and 2E). Both DR and dwarfism caused predominantly gains in DNA methylation, whilst rapamycin treatment induced equal amounts of hyper- and hypomethylated DMRs (Fig 2D and 2E). Remarkably, 412 hypermethylated DR-related DMRs (33%), and 133 hypermethylated rapamycin-related DMRs (17%) were also detected in Ames dwarf mice (Fig 2F). In addition, 9 hypermethylated DMRs were common among all three interventions. Given that the global methylome remained unchanged, these overlaps were highly significant (each: *p* < e^-16^, Fisher’s exact test), strongly indicating that their co-occurrence was not due to chance. In clear contrast, we did not detect any hypomethylated DMRs common to all three interventions and only few hypomethylated DMRs between any two interventions, for which we found no convincing functional association (Fig 2G).

**Fig 3:**
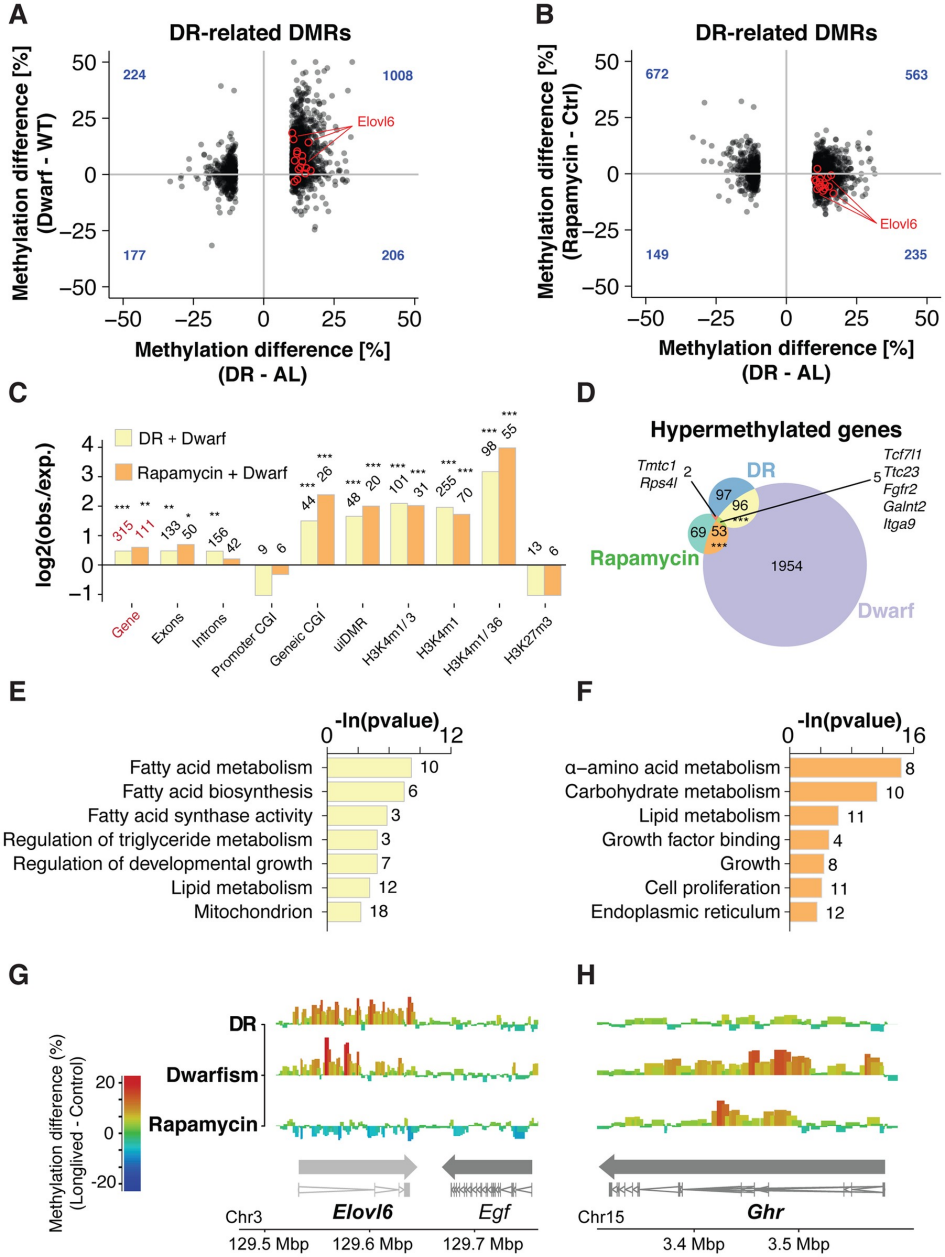
Conserved intervention-related remodeling of gene body methylation. (A, B) Scatterplot comparison of bin-wise methylation differences between DR-related DMRs versus changes in the same bins in Ames dwarf (A) and rapamycin-treated (B) mice, respectively. Bins overlapping the Elovl6 gene are highlighted in red. Methylation differences under DR were significantly positively associated with differences in Dwarfs (One-sided *Fisher’s exact* test p <0.001) but not with rapamycin-treated mice. Number of bins in each quadrant is indicated in blue. (C) Enrichment analysis of common hypermethylated DMRs between DR and Dwarfs (n = 403) and rapamycin treatment and Ames dwarf mice (n = 124) over genomic and ENCODE chromatin segmentation. Bars indicate the ratio of the observed DMR frequency and the average frequency across the genome (log2-transformed; adjusted p-values; *** p<0.001, ** p<0.01, * p<0.05, *Fisher’s exact* test). (D) Venn diagram depicting the overlap of hypermethylated genes (≥ 2 DMRs overlapping) under DR, dwarfism and rapamycin treatment (p-values; *** p<0.001, ** p<0.01, * p<0.05, *Fisher’s exact* test). (E, F) Functional enrichment of common hypermethylated genes between Ames dwarf mice and DR (E; n = 101) or rapamycin-treatmed mice (F; n = 58). (G,H) Differential methylation landscape of the Elovl6 (G) and Ghr (H) gene loci. Bins are represented as bars with color scale and height indicating methylation differences. Arrows indicate gene orientation; merged mRNA structure is depicted below.

The relatively small overlap of hypermethylated DMRs between DR and rapamycin-treated mice could partly result from low read coverage, which reduces the statistical power. To evaluate this possibility, we conducted a less constrained analysis and asked whether significant DR-related DMRs would associate with methylation changes in Ames dwarf or rapamycin treated mice, independent of their statistical significance (for details see “Methods”). DR-related DMRs associated with methylation changes in Ames dwarf (*p* < 0.001, one-sided Fisher’s exact test) but not in rapamycin-treated mice (Fig 3A and 3B), confirming our earlier result.

Finally, we considered that the relative large absolute bin size (~ 4 Kbp) might reduce the detectability of common methylation patterns and thus repeated the binning and differential methylation analysis with bins covering only 20 CpGs (2.3 mio. bins; median size: 1.49 Kbp) and 10 CpGs (4.6 mio. bins; median size: 649 bp) (S2A Fig). Consistent with the 50-CpG binning, the majority of DMRs under DR feeding and in Ames dwarf mice tended to be hypermethylated, and genetic dwarfism caused more DMRs than DR or rapamycin treatment (S2B Fig). Reducing the number of CpGs per bin increased the overall noise between long-lived and control animals (S2C Fig), yet the overlaps among the hypermethylated DMRs remained largely consistent (S3A Fig) and common 10-CpG-DMRs mapped to the same regions detected with 50-CpG bins (S3B Fig). Remarkably, we did not detect any hypomethylated DMRs common to all three interventions, thus validating our previous findings (S3C Fig).

Taken together, reduced growth factor signaling and reduced TOR signaling induce DNA methylation signatures in distinct regions of the genome, pointing to a similar epigenetic remodeling as identified before under DR. In addition, several hundred hyper- but not hypomethylated DMRs are common to at least two interventions, indicating a partly conserved epigenetic signature across interventions, mouse strains and sexes.

### Ames Dwarf mice share functionally distinct gene body methylation patterns with rapamycin-treated and dietary restricted mice

Following our findings from DR-related hypermethylation of lipid metabolism genes, the overlapping hypermethylation signatures among interventions could suggest a partially shared epigenetic program, acting on specific, functionally connected regions of the genome. In line with our hypothesis, hypermethylated DMRs shared between Ames dwarf and either DR or rapamycin treatment were strongly enriched over gene bodies and open/active chromatin, confirming coordinated methylation increase over euchromatic, coding regions of the genome (Fig 3C; Chromatin segmentation data from the ENCODE project [60]). To assess if specific functional elements within genes were particularly associated with intervention-related methylation changes, we repeated the enrichment analysis using the 10-CpG-DMRs (S4A–S4C Fig) for improved resolution. We found equal enrichment of common intervention-related DMRs over exons, intron and exon/intron-boundaries, thus confirming that these hypermethylation patterns tend to equally affect each part of the gene body (S4D Fig). Using annotation from the ENCODE and Cistrome platforms [61], we also confirmed a strong enrichment of binding sites of several transcription factors and DNA-binding proteins (S4B and S4C Fig).

As described before [49], hypermethylated genes under DR were functionally enriched for fatty acid metabolism and regulation of lipogenesis (S5A Fig; S6 Table). In contrast, rapamycin treatment caused hypermethylation primarily over genes involved in carbohydrate and amino acid metabolism, and several cell proliferation and growth factor receptor genes (S5B Fig), including mediators of TGF-β signaling (*Acvr2b* [62] *Ltbp1*, *Ltbp4* [63]) and the growth hormone receptor *Ghr* (S5B Fig; S7 Table). Differentially methylated genes in Ames dwarf mice exhibited the strongest functional association, with enrichment for lipid synthesis, amino acid metabolism, wound healing, cellular differentiation and growth factor binding (S5C Fig; S8 Table). Genetic dwarfism thus appeared to cause hypermethylation over similar pathways as observed under DR- and rapamycin-treatment separately.

We therefore analyzed the functional association of genes that were hypermethylated in at least two interventions (≥ 2 overlapping DMRs per intervention). Indeed, Ames dwarf mice shared hypermethylation patterns over lipid metabolism genes with DR fed mice (Fig 3D and 3E; S10 Table), while also showing hypermethylation of cell proliferation and growth factor signaling genes similar to rapamycin-treated mice (Fig 3D and 3F; S11 Table). As exemplified by *Elovl6* and *Ghr* (Fig 3G and 3H, S4D Fig), DNA methylation was altered over larger regions of the gene body, similar to findings in both DR cohorts (Fig 1J–1K). In summary, the non-genetic interventions DR and rapamycin treatment thus resemble different components of the epigenetic program induced by genetic dwarfism. Interestingly, dwarfism reduces expression of genes involved in hepatic lipogenesis [64,65] and alters expression of genes involved in growth factor signaling and cell proliferation [64,66], thus paralleling the observed methylation signatures.

### Hypermethylation patterns associated with longevity-extension are not explained by retardation of ageing alone

DR, rapamycin treatment and reduced somatotropic signaling slow down age-related changes of the methylome [47–50,52,67]. The common hypermethylation patterns described here were analyzed in old animals and could thus result from protection of specific genes against age-related methylation changes. Alternatively, common hypermethylation DMRs may have formed by an active reprogramming that is partly unrelated to ageing. To assess whether longevity intervention-related DMRs resulted solely from delayed ageing, we first profiled age-related changes in wild type, normally fed, non-drug-treated members of each of the three mouse strains (S6A Fig). Pre-processing and binning based on young and old control samples across strains yielded 859,750 bins, showing globally only mild age-related changes (S6B Fig). Ageing induced only 3,349 and 3,133 DMRs in C3B6F1♀ and UM-HET3♀ mice, respectively, but over 8,500 significant changes in the genetic background of the Prop1♂ strain, suggesting a strong interaction between genotype and age on DNA methylation (S6C Fig; S12 Table). Age-related DMRs in the genetic background of the Prop1♂ mice furthermore showed a bias towards age-related hypomethylation (S6C and S6D Fig), which was not observed in the other two wild type mouse strains. In order to minimize these strain-specific biases, we selected age-related DMRs present in at least two strains (S6E and S6F Fig) as reference for a comparison with intervention-related DMRs. Strikingly, 282 of 545 (53%) intervention-related, hypermethylation DMRs (found in at least two interventions) were not hypomethylated during ageing (S7A Fig; S5 Table). This result suggests that intervention-related methylation changes were not solely a consequence of general protection against ageing-related changes in the epigenome and instead reflect a specific feature of longevity interventions.

### Intervention-related DNA methylation is rapidly induced at young age

We next determined whether intervention-related methylation changes were already detectable at young age. We analyzed DNA methylation of young DR-treated C3B6F1♀ (5 months) and Ames dwarf mice (2 months) in comparison to their respective controls (S3B; data for young rapamycin- or DR-treated UM-HET3♀ mice were unavailable). Our binning approach yielded 1,018,051 bins covering 25.5 million CpGs. Differential methylation analysis detected 1,944 DR- and 5,547 dwarfism-related DMRs at young age (S13 Table), with similar hyper-to-hypomethylation ratios as observed in old animals (compare Fig 2D, S7C Fig). DR- and dwarfism-induced methylation changes at young age were also significantly associated with each other, confirming common epigenetic patterns already at this age (S7D Fig). Strikingly, intervention-related, hypermethylated DMRs in young animals were significantly enriched over intervention-related DMRs that were also detected at old age. Reprogramming of the epigenome in response to DR and reduced somatotropic signaling is thus induced in young adult animals, prior to age-related methylation changes (S7E Fig).

We further asked if intervention-related DNA methylation patterns in young mice also affected the same genes as seen at old age and found a weak but significant overlap (12%) between young and old DR fed mice (S7F and S7G Fig), including lipid metabolism genes *Elovl6*, *Scd1* (S7H and S7I Fig) and *Pnpla3*. The epigenetic reprogramming under DR is thus induced early in adulthood, but becomes more pronounced during chronic long-term treatment. In contrast, the set of common hypermethylated genes between young and old Ames dwarf mice covered over 650 genes (69% overlap), revealing that the extensive epigenetic reprogramming in response to reduced somatotropic signaling occurs early in life and is maintained life-long (S7G Fig). DR and reduced somatotropic signaling thus remodel the epigenome before age-related changes occur, additionally confirming that hypermethylated patterns detected in long-lived mice cannot solely result from deceleration of ageing.

### Joint BS-seq profiling identifies candidate marker regions of longevity

While reliable DNA methylation markers (‘epigenetic clocks’) for murine ageing have been previously developed, including local markers like *Elovl2*, a specific epigenetic biomarker predicting interventions that will ameliorate ageing in mice does not exist [46–48,68]. Such a marker would probe immediately for responsiveness to interventions, instead of wait for the delay of age-related effects. The WGBS-seq profiles analyzed here comprised three different interventions in different genetic backgrounds, thus representing a suitable dataset to investigate the general feasibility of an intervention-related marker. We found nine hypermethylated DMRs shared among all three longevity interventions (see S7A Fig; S5 Table), as exemplified by the *Tat* gene locus (S8A and S8B Fig). Of these, four hypermethylated DMRs did not overlap with age-related hypomethylation (S7A Fig; S5 Table) making them suitable marker candidates to probe for longevity-intervention-specific effects. DNA methylation levels of individual cytosines present within the four DMRs increased in concert in all three longevity interventions (p<0.001, Paired *Wilcoxon rank-sum* test) (S8C Fig). We independently tested the putative marker regions in DR-treated UM-HET3 mice, which were not used to identify these regions previously, and found the expected hypermethylation pattern (p<0.001, Paired *Wilcoxon rank-sum* test) (S8C Fig).

Next, we reduced the DMRs to four representative 500 bp regions that can be investigated by PCR-based assays. We confirmed that hypermethylation of the 125 resulting cytosines was still well correlated for all three interventions (S9A Fig; S14 Table). We also detected significant hypermethylation of these cytosines in DR-treated UM-HET3 mice (S9A; p<0.001, Paired *Wilcoxon rank-sum* test). The four regions were selected to not overlap with age-related changes in DNA methylation (S7A Fig) and accordingly, we did not observe consistent methylation changes with age for the representative 500 bp regions (S9B Fig).

We asked if the putative marker regions would be able to predict extended lifespan when analysed in young DR or Ames dwarf mice. Indeed, young Ames dwarf mice showed significant hypermethylation over the marker cytosines, indicating that reduced somatotropic signaling during development primes for an extended lifespan (S9C Fig). Our marker readout is thus in agreement with analyses of the epigenetic clock in young Ames dwarf mice, which is already significantly decelerated at 2 months of age [47]. Surprisingly, however, young DR fed C3B6F1♀ mice (3 months ad libitum feeding followed by 2 months DR) showed no significant difference over the marker regions. The marker thus indicates that 2 months of DR feeding at young age do not suffice to extend lifespan later on (S9C Fig). Indeed, recent studies in rats confirmed that DR feeding limited to two to six months of age does not cause an extension of lifespan later on [12]. This result is also consistent with findings from DNA methylation clocks, where long-term, but not short-term DR, resulted in a significant reduction of DNA methylation age [48]. In addition, we tested whether the marker regions could also be used in other tissues and analysed the RRBS-seq data from blood of DR fed B6D2F1♂ mice (see S1 Fig). Only half of the 125 CpGs were covered by RRBS-seq and the corresponding methylation levels in blood of control animals did not match the observed levels in the liver (compare S9A and S9D Fig). However, despite the low coverage there was no evidence for a predictive function of this marker region in the blood.

In order to verify our marker experimentally, we measured DNA methylation at the marker regions in five DR and five AL fed C3B6F1♀ mice (24 months of age) from an independent batch of animals using targeted Bisulfite Amplicon sequencing (BS-AS; for details see “Methods”) [69]. To test if the marker would probe successfully for other lifespan-extending interventions, we also performed BS-AS in four female C3B6F1 mice genetically deficient for Irs1 (Irs1KO; 24 months of age) [70] and four controls. The BS-AS assay measured DNA methylation levels at 80 CpGs, thus covering most of the CpGs within the marker regions (S10A Fig). Unsupervised hierarchical clustering of methylation levels at these 80 CpGs separated most of the samples into controls and long-lived mice, indicating that DNA methylation at these sites can classify for extended lifespan (S10B Fig). Averaged methylation levels across all replicates clustered similarly, confirming that they are not biased by single outlier replicates (S10B Fig). Remarkably, we detected a significant concerted hypermethylation at these CpGs both in DR fed and Irs1KO mice (S10C; p<0.05, Paired *Wilcoxon rank-sum* test). Consistently, we found 18 hypermethylated dCpGs under DR feeding and 32 dCpGs in Irs1KO mice (4 and 12 hypomethylated dCpGs, respectively). One of the four marker regions, however, appeared to be false positive in the WGBS-seq data, as most long-lived animals showed no consistent change at these CpGs (S10B Fig). Excluding the corresponding CpGs improved the result of the detection of concerted hypermethylation (S10D; p<0.05, Paired *Wilcoxon rank-sum* test).

In summary, concerted hypermethylation of these marker cytosines in liver correlates specifically with longevity upon DR, reduced TOR and reduced growth factor signaling. By testing the marker cytosines in an independent model of longevity, we demonstrate the feasibility of developing novel epigenetic markers that can probe specifically for intervention outcome.

### Epigenetic reprogramming parallels transcriptional repression of growth factor signaling networks in Ames dwarf mice

**Fig 4:**
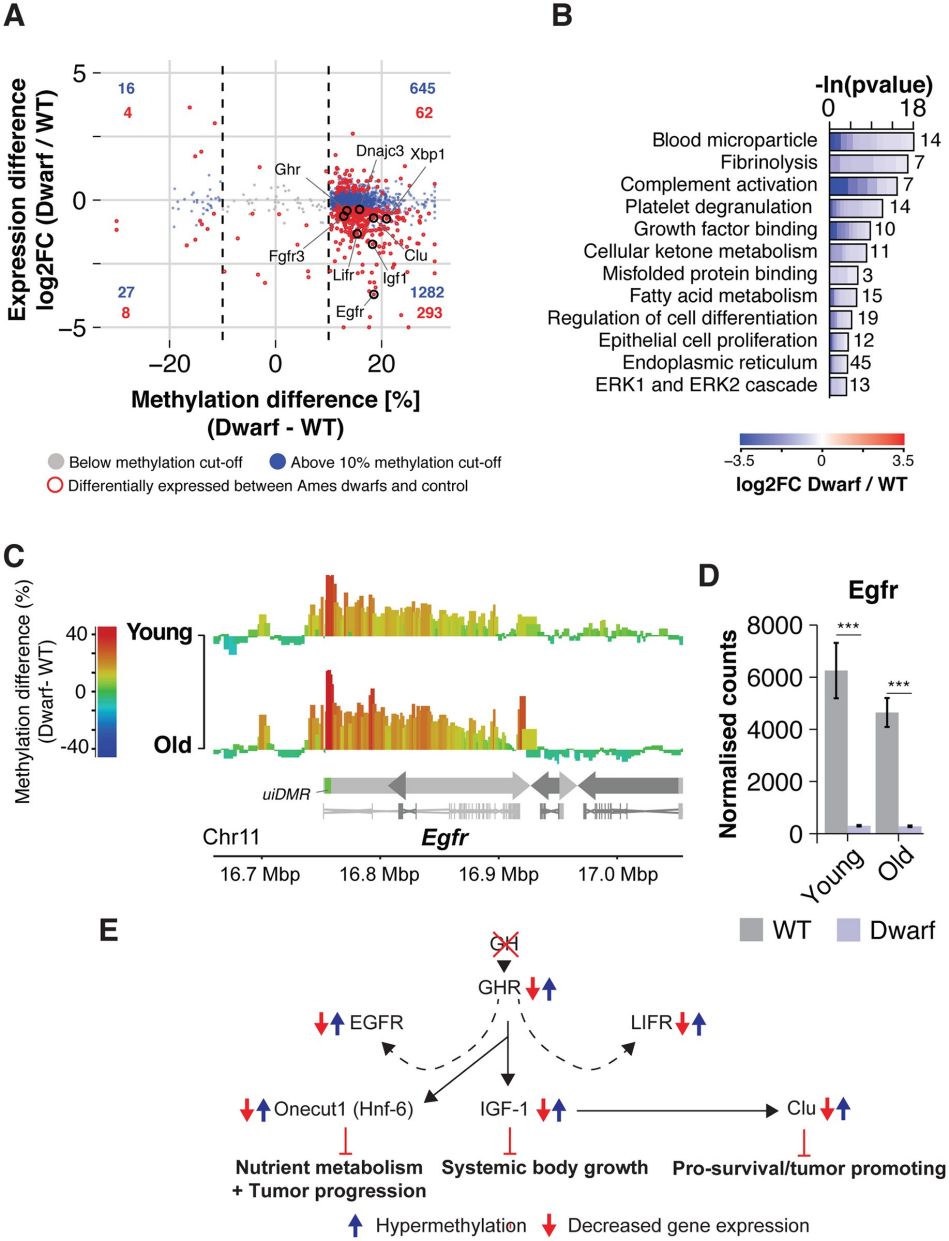
DNA methylation differences in Ames dwarf mice predict expression changes. (A) Scatterplot of expression differences versus methylation differences of dwarfism-related DMRs. Dashed lines indicate DNA methylation cutoff of > ±10%. DNA hypermethylation associated significantly with reduced gene expression (*Binomial* test p < 0.001). Number of differentially methylated genes in each quadrant is indicated in blue and red, for all genes and differentially expressed genes, respectively. (B) Gene ontology and Reactome enrichment of genes with a negative correlation of gene expression and methylation. Lengths of bars represent negative ln-transformed, adjusted pvalues using *Fisher’s exact* test. Cells indicate log2-foldchanges (log2FC) between Ames dwarf and control group per gene. (C) Differential methylation landscape of the Egfr gene locus at young and old age. Bins are represented as bars with color scale and height indicating methylation differences. Arrows indicate gene orientation; merged mRNA structure is depicted below. (D) Egfr mRNA expression by RNA-sequencing in control and Ames dwarf mice at young and old age, respectively (n = 4 vs 2 and 4 vs 4). (E) Schematic outline of differential methylation and gene expression along the Hypothalamic–pituitary–somatotropic axis in Ames dwarf mice: In Ames dwarf mice, the release of Growth Hormone (GH) is impaired. The corresponding Growth hormone receptor (*Ghr*) shows respective transcriptional downreguation in the liver. Direct (solid arrows) and indirect (dashed arrows) downstream targets of GHR signaling, show elevated methylation levels and corresponding transcriptional repression. Further hypermethylated/downregulated targets include the hepatoprotective factors *Egfr*, *Lifr* and *Onecut1*, involved liver growth regulation, cell survival and metabolism.

Gene body hypermethylation under DR has been associated with transcriptional repression of lipogenesis, pointing to a tight coupling between DR-related remodeling of the epigenome and gene expression [49]. We asked if expression and differential methylation would also be inversely associated in Ames dwarf mice, given their similar epigenetic patterns to DR-treated mice. Focusing on genes with at least 2 dwarfism-related DMRs mapping to the gene body, we plotted methylation differences against expression changes (Fig 4A; S15 Table). As previously described for DR [49], the expression of hypermethylated genes tended towards down-regulation in Ames dwarf mice (*p* < 0.001, Binomial test) and this was even more pronounced when considering only significantly differentially expressed genes (Fig 4A; S15 Table). We also found this inverse correlation to be present in young Ames dwarf mice (S10A Fig; S16 Table), suggesting this association to be early-onset and maintained life-long. Correspondingly, we found at young and old age similar functional enrichments of GO terms and Reactome pathways, including Unfolded Protein Response (UPR), lipid metabolism and wound healing (Fig 4B, S10B Fig; S16, S17 and S18 Tables). In addition, we found strong dwarfism-related hypermethylation and repression of several growth factor signaling and cell survival genes (Fig 4B–4D, S10B and S10C Fig; S16, S17 and S18 Tables). Notably, this included the growth hormone receptor (*Ghr*, Fig 3H) and several direct and indirect downstream targets, such as the systemic growth mediator *Igf-1* [71] and the hepatoprotective genes *Egfr*, *Lifr* and *Onecut1* (Hnf-6) [72–78]. These regulators of cell survival and growth carry out key functions in liver regeneration and also play major roles in promoting cancer onset and progression [79–87].

Whilst increased DNA methylation in gene bodies is typically correlated with increased expression [88], recent studies also demonstrated an inverse association [89–91]. For instance, the strongest inverse association between DNA methylation and expression in humans was previously found in so-called undefined intragenic DMRs (uiDMRs), which locate 0.3–8 Kbp downstream of the promoter. [89]. In line with these findings, we observed an enrichment of intervention-related methylation changes at uiDMRs (Fig 3C and S4A Fig). Moreover, the significant inverse association between dwarfism-related changes in methylation and expression remained consistent when restricting the analysis to uiDMRs (S10D and S10E Fig). Differential methylation over uiDMRs may thus be a putative mechanism mediating the transcriptional consequences of the epigenetic reprogramming.

In summary, our results thus reveal strong epigenetic and transcriptional silencing of the growth hormone signaling axis, which may contribute to delayed occurrence of neoplastic diseases in Ames dwarf and growth hormone signaling deficient mice [9,29,30].

### Dwarfism and DR remodel methylation and expression of Fgfr3

Given the common epigenetic signatures occurring across longevity interventions (Fig 2E and 2F), we asked whether methylation and transcriptional changes would also be common between DR fed and Ames dwarf mice (RNA-seq data for rapamycin-treated mice were not available). In line with previous work [49], we found hypermethylation and transcriptional repression of lipogenesis genes to be significantly associated in DR fed mice (*p* < 0.05, Binomial test; S11A and S11B Fig; S19 and S20 Tables). DR and Ames dwarf mice showed similar transcriptional patterns, with an overlap of 421 commonly regulated genes (S11C and S11D Fig). Surprisingly, the overlap between commonly hypermethylated and significantly repressed genes comprised only a non-significant set of five genes, suggesting that DNA methylation and transcription are differently coupled under DR and reduced somatotropic signaling (S11E Fig). Interestingly, however, fibroblast growth factor receptor 3 (*Fgfr3*, S11F–S11H Fig), a key mediator of mitogenic signals and known activator of both the *Ras*-*Mapk* and *PI3K-AKT* pathways [92], was found to be hypermethylated and repressed in DR and Ames dwarf mice.

As a crucial regulator of cell growth and survival, *Fgfr3* is frequently up-regulated in various cancers including hepatocellular carcinoma [93–96]. Hepatic *Fgfr3* is also repressed in mouse models of genomic instability, which exhibit dwarfism and GH-IGF1-signaling deficiency, thus paralleling our findings in Ames dwarf mice [97,98]. Hence, the anti-tumorigenic effects of DR and dwarfism [9,29] may involve repression of *Fgfr3*. Furthermore, *in-vitro* studies with bladder cancer cell lines confirmed *Fgfr3* as a direct activator of lipogenesis via *Srebf1* [99–101], which itself becomes hypermethylated and repressed under DR [49,102,103]. Remarkably, gene expression changes induced by knockdown of *Fgfr3* in RT112 cells [100] overlapped significantly with expression changes observed in livers of DR fed C3B6F1♀ mice (S12A–S12C Fig; S21 Table) including down-regulation of various hypermethylated lipogenesis genes (S22 Table). Our analysis thus point to a role of *Fgfr3* in mediating the effects of DR and genetic dwarfism in the liver.

## Discussion

Physiological consequences of DR including lifespan extension are strongly dependent on genetic background, sex and protocol [10,22,104]. After detecting epigenetic reprogramming of lipid metabolism in response to DR in an earlier study [49], we aimed to determine the robustness of these DNA methylation changes in a different genetic background. We further investigated the extent to which the epigenome is also remodeled in response to other longevity-extending interventions. We hypothesized that this epigenetic remodeling might occur independently of decelerated ageing effects. In this case, intervention-related methylation patterns would (I) show a pattern distinct from age-related methylation changes and (II) already be detectable in young animals, i.e., before age-related changes can occur. Given that epigenetic modifications mediate long-term consequences for gene expression [40,105], remodeling of the methylome could causally contribute to improved health at old age and increased lifespan. We therefore investigated a possible association between intervention-related methylation and gene expression.

The net effect of DR on the methylome was highly comparable between strains and studies. Interestingly, the total number of DR-related DMRs in UM-HET3♀ mice was about 56% higher compared to C3B6F1♀ mice, which correlates with the ~56% stronger improvement in lifespan in the genetically heterogeneous strain (+39% in UM-HET3♀ and +25% in C3B6F1♀mice, relative to control). This may hint towards an association between the extent of the epigenetic reprogramming and the improvement in lifespan. However, when comparing which specific regions were affected, approximately 80% of DR-related, hypermethylated DMRs and 95% of hypomethylated DMRs were only detected in one mouse strain/dataset. While these differences may be, in part, due to the genetic background and differing sequencing protocols [106], there were other confounding factors between the studies. For instance, the food composition differed between both DR experiments, which is known to affect metabolic outcomes of DR [24]. This may in turn affect the DR-induced epigenetic changes, and consequently the comparability between the two strains. Consistently, we observed a stronger cross-strain conservation of methylation patterns in blood of closely related, DR-fed mouse strains from the same study, i.e. that were housed at the same animal facility and undergoing identical sample preparation/sequencing [48]. Given our conservative filtering, common DMRs in the liver may thus not reach statistical significance in two separate datasets. In this context, the 20% overlap of hypermethylated DMRs represents a remarkably robust core set of DR-related DMRs.

Hepatic mRNA expression changes between two commonly housed, DR-fed mouse strains showed a 50% overlap in their response to DR [10], indicating that DR induced gene expression changes might be better conserved between mouse strains than epigenetic changes. Interestingly, this and other studies identified suppression of hepatic lipogenesis as one of the most conserved transcriptional signatures of DR in rodents [10,49,102,103,107], which parallels the conserved hypermethylation events affecting direct targets of the lipogenic transcription factor Srebf1, including *Fasn*, *Acly*, *Me1*, *Elovl6* and *Scd1* [55,108–112]. Our findings thus link core DR-related expression patterns with their directed epigenetic reprogramming over time.

Our analysis extended the concept of intervention-related DNA methylation changes to both reduced TOR and somatotropic signaling. We also found here that the intervention with the strongest improvement in lifespan (dwarfism) induced the most DMRs, while rapamycin and DR, which have similar effects on lifespan, induced comparable numbers of DMRs. However, the detection of significant methylation changes is dependent on various technical factors such as coverage [106], and we can therefore not exclude that these correlating observations are rather coincidental.

Hypomethylated DMRs were only shared to a minor extent among interventions, and we found no convincing functional association. Hypomethylated DMRs could thus represent treatment-specific changes. In contrast, intervention-related, hypermethylated DMRs were commonly targeted to gene bodies and partly shared among interventions. Interestingly, downstream mediators of the key longevity pathways, PI3K/Akt (*Gsk3β*) and Raf signaling (*Erk1*/2) [1,2,113,114], regulate de-novo methyltransferase 3b (*Dnmt3b*) [115], which may be explored as potential mechanism driving the formation of common hypermethylation patterns. Around 65% (DR) to 95% (Ames dwarf mice) of hypermethylated DMRs were only found in one intervention. However, given the results from the cross-strain comparison of DR-related effects, about 80% of non-common DMRs can be expected by differences in genetic background and technical variation alone. Sex differences in patterns of DNA methylation [116,117] may further reduce the comparability between epigenomes. We cannot yet test directly for the importance of these experimental factors in a meta-analysis, but presumably the set of common DMRs would have been greater if all the experiments had been conducted with identical conditions and protocols.

Notwithstanding, methylation over four regions robustly increased in response to all three interventions independent of strain and sex, while showing no age-related changes, thus meeting the main requirements for a putative epigenetic marker to directly assess the response to lifespan-extending interventions. As such, we could experimentally confirm the validity of our marker in an independent batch of mice and successfully probe for longevity in Irs1KO mice that were not involved in the marker selection. Albeit weak in effect, methylation at the CpGs measured by targeted BS-AS was overall sensitive enough to detect the anticipated, significant difference. Epigenetic clocks [42,46–48] and methylation-based biomarkers of biological age [68,118] measure the ‘ticking rate’ of ageing and can therefore probe only for the indirect deceleration by longevity interventions. In addition, mouse epigenetic clocks currently rely on sufficient coverage of all clock-CpGs by RRBS-seq and show error rates of up to ± 4 months [47,48], which limits assessment of short-term effects.

In contrast, concerted hypermethylation over just these four regions was reliably correlated with longevity intervention outcome. None of the clock-CpGs mapped within the four intervention-related DMRs [46–48], unsurprising since clock-CpGs were selected to probe ageing. In addition, one of the four DMRs mapped to the *Tat* gene locus, which has been previously shown to be unaffected by age-related changes in DNA methylation in rat liver, spleen and brain [119]. The putative marker regions thus seem truly sensitive to intervention- but not age-related effects. We found no obvious functional association between the DMRs, of which two mapped over genes (Exon of *Tat*; intronic CGI of *Foxp4*) and two at intergenic regions, suggesting that methylation markers are not directly linked to a distinct cellular process [42,46–48]. The marker regions presented here could be easily analyzed in published WGBS-seq datasets or directly assessed by with our BS-AS protocol, thereby reducing costs and experimental effort.

Our findings act as a proof-of-principle to demonstrate the general feasibility of a treatment-specific DNA methylation marker. Due to the limited number of available samples, the marker DMRs yield only a binary output (statistically significant or non-significant hypermethylation). It will be interesting to test the corresponding methylation patterns in lifespan-extending interventions that do not primarily target energy metabolism, e.g. aspirin treatment [120] or ablation of senescent cells [121]. Finally, we show that the marker region appears to be dependent on the tissue, which, in the case of the liver tissue used in this study, could only be sampled by invasive techniques, and similar markers should be developed for blood or skin. Future marker assays would allow defining the critical treatment length and timing for new longevity interventions to improve lifespan, and assess the sustainability of the effects once the intervention is suspended.

In agreement with common signaling pathways associated with the three lifespan-extending interventions [15,18,19,34–37], we found that DR and rapamycin treatment resembled functionally distinct epigenetic patterns of Ames dwarf mice. Metabolically, dwarfism protects from hepatic insulin resistance, reduces high-fat diet induced liver steatosis and suppresses de-novo lipogenesis genes including *Elovl6*, *Acly* and *Fasn* [64,122,123], thus indicating reduced lipogenic activity through Srebf1 [108,124–126]. Snell dwarf mice, another mouse model of reduced somatotropic signaling, also show age-independent down-regulation of *Srebp1* and several downstream targets in the liver [127]. Furthermore, DR fed mice and Snell/Ames dwarf mice exhibit altered transcriptional dynamics of peroxisome proliferator-activated receptor α (PPARα) [128], which in turn regulates Srebp1 activity [129,130] and may thus explain the similar expression patterns of lipogenesis genes. Consistently, in both DR and Ames dwarf mice we observed hypermethylation over several Srebf1-target genes (*Elovl5*, *Elovl6*, *Fasn*, *Gpam*, *Acly*, *Acaca*, *Aacs*) [55,108,112,131–134]. Their hypermethylation was further accompanied by transcriptional repression in Ames dwarf mice at young age, which was also observed in young Snell dwarf mice [127]. Hence, a conserved epigenetic module links metabolic reprogramming with increased protection against hepatic steatosis and insulin resistance in Ames dwarf and DR mice. Rapamycin, in contrast, activates hepatic lipid metabolism [135,136] and shares only a small subset of regulated transcripts with DR [137], which is in line with hypomethylation of genes involved in lipid and fatty acid metabolism (compare S5A and S5B Fig). These opposite methylation changes might also explain the higher number of hypomethylated DMRs under rapamycin treatment and the weak overlap with DR.

Pharmacological suppression of mTOR affects cellular sensitivity to growth factors [138], which also marks a fundamental consequence of genetic dwarfism [28]. Rapamycin represses growth factor signaling during liver injury and fibrosis [66,139] and is tested in clinical trials for the treatment of advanced hepatocellular carcinoma (HCC) [140], indicating a role in both tissue regeneration and cancer. Consistently, hypermethylation of growth factor receptors/mediators such as *Lifr* [78], *Fgfr2* [141], *Acvr2b* [62], *Ltbp4*, *Ltbp5* [63] and the Growth hormone receptor (*Ghr*) suggests long-term adaptation of the epigenome to altered growth and mTOR signaling, which was even more prevalent in Ames dwarf mice.

In Ames dwarf mice specifically, we show for the first time how reduced somatotropic signaling is paralleled by potent hypermethylation and transcriptional repression of the Ghr-Igf1 axis and downstream target genes with key roles in tissue growth and regeneration [72–76]. These hepatoprotective mediators (*Egfr*, *Lifr*, *Onecut1*) [78] are also highly oncogenic [72,73,78,79] and tight control of their expression could therefore contribute to the delayed onset of neoplasia in Ames dwarf mice [29,30]. The age-ameliorating effects of dwarfism have also been implicated in the prevention of cancer by maintaining a youthful epigenome [50], which may work in tandem with the epigenetic reprogramming of mitogenic signaling described here. Thus, Ames dwarf mice instigate an ample epigenetic silencing of growth hormone-related signaling, which may also help to delay the onset of cancer.

The remarkably strong association between intervention-related shifts in DNA methylation, transcription and phenotype marks a key difference to age-related methylation changes, where epigenome and gene expression changes are generally not correlated [49,50,142]. Age-related changes also tend to affect predominantly promoter CGIs [46,49], while intervention-related DMRs, which are not a result of delayed ageing alone, tend to map over intragenic elements. Given the strong link between robust intervention-induced expression [143,144] and the gene body methylation patterns in long-term DR and Ames dwarf mice, it is thus tempting to speculate about a direct relationship. As such, we have identified a strong inverse association between hypermethylation at uiDMRs and mRNA expression, and elucidating a potentially causal connection will be an interesting task for future studies. In addition, increased DNA methylation has been suggested to reduce Pol-II elongation speed [145,146] and thus could further explain the associated transcriptional repression.

However, gene expression involves an intricate time- and context-specific interplay of epigenetic mechanisms and transcription factors. Genes that show consistently coupled methylation and expression changes in both DR and Ames dwarf mice are therefore strong candidates for further exploration given their robust regulation. For instance, Fgfr3 is an oncogenic mediator of mitogenic signals [94,96,147] and activator of Srebf1-mediated lipogenesis [99–101]. Down-regulation of Fgfr3 could thus be a candidate mechanism for exploring the signaling effects of both dwarfism and DR. Critically, *in-vitro* knockdown of Fgfr3 not only mimicked the DR-like repression of lipogenesis, but also DR-induced up-regulation of transcriptional regulators like *Jdp2* [148], *Nfkbia* [149] and *Arnt* [150]. Epigenetic reprogramming of Fgfr3 could therefore exert extensive downstream effects, which may contribute to improved health during ageing.

### Conclusion

This study provides novel insights into the effects of longevity interventions on DNA methylation and how they may causally contribute to improved health at old age. Dietary restriction (DR), rapamycin treatment and reduced somatotropic signaling remodel DNA methylation partly independent of ageing, converging on defined marker regions predictive of intervention outcome. In addition, DR and rapamycin treatment resemble separate hypermethylation patterns of Ames dwarf mice, affecting lipid metabolism and growth factor signaling genes, respectively. For DR and Ames dwarf mice, we furthermore demonstrate significant association between DNA methylation and gene expression. Therefore, our work establishes hypermethylation signatures as a link between the long-term effects of several longevity models and their transcriptional networks controlling somaotropic signaling and metabolism.

## Methods

### Ethics statement

The study involving live mice was performed in strict accordance with the recommendations and guidelines of the Federation of the European Laboratory Animal Science Association (FELASA), with all protocols approved by the Landesamt für Natur, Umwelt und Verbraucherschutz, Nordrhein-Westfalen, Germany (reference number: 84–02.04.2015.A437). For dissections mice were killed by cervical dislocation.

### Bisulfite Amplicon sequencing

Bisulfite Amplicon sequencing (BS-AS) libraries were prepared from liver tissue of 24 months old female C3B6F1♀ mice with the following treatment groups: 5 DR or 5 AL fed mice (for animals husbandry see [49]), 4 homozygous IRS1-mutant and 4 wild-type mice. DNA was isolated using the DNA/RNA Allprep kit (Qiagen) and bisulfit conversion was done using the Imprint DNA modification kit (Sigma) following the one-step modification procedure. The KAPA HiFi Uracil+ Master Mix kit (Kapa Biosystems) was used to amplify the 4 regions of interest using the primers indicated in S23 Table. A single round of PCR amplification was used per sample whereby the forward primer contained a 6bp sample barcode. The PCR program was: 95C 5min; 35 repeats of 98°C 20s, 60°C 15s, 72°C 60s; 72°C 10min. PCR products were purified using Ampure XP beads (Agencourt), analyzed on a 4200 Tape station system (Agilent) and subsequently pooled for library preparation. Two libraries were generated with 40 and 32 samples for the AL vs. DR and IRS-1 vs. wild-type comparison, respectively. Sequencing was performed using 250bp paired end reads on an Illumina HiSeq2500.

### WGBS-seq quantification and differential methylation analysis

An overview of the analyzed datasets and treatment conditions is given in (S1 Table). Raw datasets were downloaded from the GEO repository under accession IDs GSE89275 and GSE92486. WGBS-seq data were preprocessed and analyzed according to [49]. Raw sequence reads were trimmed using Trim Galore! (v0.4.2). Trimmed sequences were aligned using Bismark [151] (v0.16.3). Methylation calls were extracted after duplicate sequences had been excluded. Data visualization and analysis were performed using SeqMonk and custom RStudio scripts. Each age and treatment group was present in biological triplicates or quadruplets. Data from replicates of the same treatment group were merged using SeqMonk’s data group option, in order to enhance coverage and detection of subtle differences. The high consistency between replicates across the used datasets has been previously verified in the original studies [49,50]. Regions with an unusually high number of observations were detected and filtered using non-overlapping 25 kb windows, followed by read count quantification and subsequent BoxWhisker filter implemented in SeqMonk with stringency >10. To achieve a fair and unbiased analysis of the methylome, we constructed windows containing 50 CpGs over the whole genome, spaced 25 CpGs apart. To demonstrate the validity of our findings independent of the window size, we partly repeated the analyses with windows containing 20 or 10 CpGs, spaced 10 and 5 CpGs apart, respectively. Each window therefore contained around the same amount of data and all windows had similar technical noise and statistical power. Furthermore, to reduce the effect of coverage differences between samples, only cytosines covered by at least three observations in all analyzed treatment groups were used for window construction, opposing the limitation for total number of bins and resulting bin size. Methylation for each window was calculated as the average of methylation for each covered CpG position. Windows that contained significantly different methylation levels (pairwise Chi-squared tests with subsequent multiple testing correction; adjusted p-value <0.05) and a minimal difference cutoff of 10% were defined as differentially methylated (DMR) [49].

Filtering of CpGs and binning was conducted for each of the following analyses separately: a) Old DR treated C3B6F1♀ or UM-HET3♀ mice including controls (Fig 1) b) old DR fed C3B6F1♀, Ames dwarf and rapamycin treated UM-HET3♀ mice, including controls (Fig 2) c) young and old C3B6F1♀, Prop1♂, UM-HET3♀ control mice (S2 Fig) d) young DR fed C3B6F1♀ and Ames dwarf mice, including controls (S3 Fig).

For the example in S2C, the methylation landscape over the *Tat* gene locus was visualized by calculating average CpG methylation levels of 500 bp 400 bp overlapping bins to improve resolution.

### Comparative DMR analysis

Significance of DMR sets common between two interventions was tested using *Fisher’s exact* test with the entire methylome as background. Due to the observed gene body-wide methylation profiles, differentially methylated genes were defined as common when overlapped by at least two DMRs in each intervention (not necessarily the identical DMRs). For comparing longevity-related DMRs with age-effects (S3A Fig), we selected every bin overlapping age-related DMRs detected in at least two strains as reference. To test if methylation differences induced by two interventions would be associated in general, we asked if significant DMRs detected in one intervention would correlate with methylation changes in another intervention, independent of statistical significance. To this end, we selected DMRs detected in DR treated C3B6F1♀ mice and plotted the observed methylation differences for the same bins in other interventions (DR, rapamycin and dwarfism). The number of bins within the four resulting quadrants was counted and directionality of differences was tested using one-sided Fisher’s exact test.

### Enrichment analyses of DMRs over genomic and epigenetic elements

For a given genomic element, we calculated the enrichment for a set of DMRs by counting the number of DMRs overlapping the element of interest and compared it to the set of background bins. One-sided Fisher’s exact test with subsequent multiple testing correction (if enrichment analysis was run over multiple elements) was performed to determine statistical significance of enrichment. Since most elements showed highly significant enrichment, we log-transformed the adjusted p-values. Ratio of the observed DMR frequency and the average frequency across the genome (‘observed/expected’) for individual elements were used to compare enrichments across elements and between DMR sets. This way, we were able compare the strengths of enrichments across elements in the same analysis.

### RRBS-seq quantification and differential methylation analysis

Raw datasets were downloaded from the GEO repository under accession ID GSE80672. Raw sequence reads were trimmed using Trim Galore! (v0.4.2). Trimmed sequences were aligned using Bismark [151] (v0.16.3). Data visualization and analysis were performed using SeqMonk and custom RStudio scripts. Data from replicates of the same treatment group were merged using SeqMonk’s data group option, in order to enhance coverage and detection of subtle differences. To reduce the effect of coverage differences between samples, only cytosines covered by at least five observations were used for the differential methylation analysis. Differentially methylated cytosines (dCpGs) were identified by pairwise Chi-squared tests with subsequent multiple testing correction (adjusted p-value <0.05) and a minimal difference cutoff of 10%. Filtering of CpGs and binning was conducted for old DR treated C57BL/6♂ or B6D2F1♂ mice including controls (S1 Fig).

### Functional enrichment analyses of differentially methylated genes

For the analyses employing WGBS-seq data, we performed functional enrichment of genes overlapped by at least two DMRs and only retained those showing at least ±10% average difference. We thereby avoided unclear cases with an equal extent of hyper- and hypomethylation occurring over the same gene. Enrichment analysis was carried out using topGO [152] and ReactomePA [153] with all genes in the genome as background.

For the analyses employing RRBS-seq data, we tested each gene for significant enrichment of overlapping dCpGs compared to all detected CpGs overlapping and only retained those with p-values < 0.05 from Fisher’s exact test (after multiple testing correction) and at least ±10% average difference.

### Marker regions of longevity analysis

We quantified the methylation for each CpG position individually that mapped within the four hypermethylated DMRs shared between all three longevity interventions but not with age-related hypomethylation (S3A Fig). CpGs covered by at least three observations in at least one analyzed treatment group was included and the methylation change across CpGs tested using paired *Wilcoxon rank-sum* test. The analysis was repeated after manually selecting one representative 500 bp regions for each of the four DMRs.

### BS-AS quantification and differential methylation analysis

Raw sequencing libraries were de-multiplexed before mapping and methylation extraction was conducted as described for WGBS-seq. De-duplication step was omitted. Data visualization and analysis were performed using SeqMonk and custom RStudio scripts. Data from replicates of the same treatment group were averaged using SeqMonk’s replicate set option. To reduce the effect of coverage differences between samples, only cytosines covered by at least three observations were used for the differential methylation analysis. Differentially methylated cytosines (dCpGs) were identified by pairwise, replicate-sensitive logistic regression tests with subsequent multiple testing correction (adjusted p-value <0.05). Methylation change across CpGs was tested using paired *Wilcoxon rank-sum* test.

### RNA-seq analysis

For young and old Ames dwarf and DR-fed C3B6F1♀ mice, RNA-seq data derived from liver tissue of the same animals used for WGBS-seq was downloaded from the GEO repository under accession IDs GSE89275 and GSE92486 (S1 Table). Two replicate samples of young Ames dwarf mice showed very high amounts of low quality reads and insufficient coverage and were therefore removed from the analysis. Raw sequence reads were trimmed to remove adaptor contamination and poor-quality reads using Trim Galore! (v0.3.7, parameters:—paired—length 25). Trimmed sequences were aligned using Tophat2 [154] (v2.0.14, parameters:—no-mixed—library-type = fr-firststrand -g 2 -p 15 -r 500—mate-std-dev 525), supplying GENCODE annotation [155] (release M9, main annotation) for improved mapping. Multi-mapped reads were filtered using samtools [156] (v1.2, parameters: view -F 0x100 -b–h). Data visualization and analysis was performed using SeqMonk, custom RStudio scripts and the following Bioconductor packages: Deseq2 [157], topGO [152], ReactomePA [153] and org.Mm.eg.db. For Figs 4B, S5B, S6B and S7C, we further used the CellPlot package (https://github.com/dieterich-lab/CellPlot). To account for tissue-specific expression, we defined all genes passing the independent filtering of Deseq2 [157] as ‘expressed’ (14154 and 13894 genes for C3B6F1♀ and Prop1♂ mice, respectively). Differentially expressed genes were determined using Deseq2´s Wald test [157]. P-values were adjusted for multiple testing. Genes were considered to be significantly differentially expressed with a p adjust < 0.05 and no cut-off for fold change was used. Unless stated otherwise, the set of expressed genes was used as background for all functional enrichment analyses involving expression data.

### Correlation analysis of methylation and transcription

To test for correlation between differential methylation and gene expression, we considered only genes overlapped by at least two DMRs. For associations between differential methylation over uiDMRs and expression, we considered only genes with their uiDMRs (0.3–8 Kbp downstream of gene start) overlapped by at least one DMR. We averaged methylation differences for multiple DMRs overlapping the same gene (uiDMR) before plotting, and only retained those showing at least ±10% average difference. We thereby avoided unclear cases with an equal extent of hyper- and hypomethylation occurring over the same gene (uiDMR). We plotted log2-fold expression changes versus methylation differences and the distribution of genes among the four resulting quadrants. Due to the very few hypomethylated genes common among longevity interventions, we focused on hypermethylated genes for statistical testing. For the group of differentially expressed and hypermethylated genes above the minimum methylation cut-off, we ran a binomial test to probe for a significant trend towards down- or up-regulation. Genes that showed a significant association of hypermethylation and transcriptional repression were analyzed for functional enrichment using topGO [152].

### Comparative transcriptome analysis for DR liver tissue and Fgfr3 knockdown in RT112 cells

To compare the transcriptional changes induced by DR with *in-vitro* knockdown of Fgfr3, we obtained a list of Fgfr3-regulated genes from pre-preprocessed microarray results from [100]. The list comprises significantly differentially expressed genes in three independent doxacycline-treated RT112 cell lines expressing doxycycline-inducible shRNAs targeting Fgfr3 (each cell line expresses another Fgfr3-shRNA). Gene expression was compared to a line expressing a control shRNA [100]. For each gene showing differential expression in Fgfr3 knockdown cells and liver tissue of DR-treated mice, we plotted log2-fold expression changes under DR (compared to ad libitum feeding) versus log2-fold expression changes under Fgfr3 knockdown (compared to control). We plotted the distribution of genes among the four resulting quadrants and tested for directionality using Fisher’s exact test.

### Definition of genomic and epigenetic elements

Gene annotation used in this study was obtained from the UCSC Genome Browser database [158]. We defined further genome annotation as follows:

*Promoter region*: Manually defined region stretching from 5 Kbp upstream and 100 bp downstream of the transcription start site.
*uiDMR*: According to [89], uiDMRs were defined as regions stretching from 300 to 8000 bp downstream of the transcription start site. Analysis was limited to genes with a total size of at least 16 Kbp.
*CGI*: Annotation based on CXXC affinity purification plus deep sequencing (CAP-seq) experiments [159] CGIs were further classified unambiguously into promoter, gene and intergenic CGIs. If a CGI overlapped both promoter and gene body, it was classified as a promoter CGI.
*Chromatin states*: Publicly available, pre-processed chromatin annotation in liver tissue based on four histone marks (ENCODE chromHMM segmentation) were obtained from the ENCODE table browser [60] and re-mapped to the GRCm38 genome using UCSC Genome Browser’s LiftOver [160]. Details about the corresponding methods can be found here: https://main.genome-browser.bx.psu.edu/cgi-bin/hgTrackUi?g=meryChromHmm7s&db=mm9).

## Supporting information

S1 Fig Dietary restriction-related methylation changes are tissue-specific. (A) Schematic representation of the data sets analysed and comparisons made. We probed for Dietary Restricted (DR)-related dCpGs in blood of adult B6D2F1♂ and C57BL/6♂. Pre-processing was conducted in parallel for all experimental groups (B) Number of dCpGs (p<0.05, ±10%< DNA methylation difference) between each DR cohort and its respective control. Proportion of hypermethylated dCpGs are indicated in %. (C) Venn diagrams depicting the overlap of significantly hypo- and hypermethylated dCpGs in both DR cohorts. Proportions of overlap relative to the C57BL/6♂ set are indicated in % (p-values; *** p<0.001, ** p<0.01, * p<0.05, *Fisher’s exact* test). (D,E) Edwards-Venn diagrams depicting the overlap of significantly hypo- (E) and hypermethylated (D) genes in liver (see Fig 1) and blood present in the four tested DR cohorts. Liver and blood datasets are indicated by solid and dashed lines, respectively.
(TIF)

S2 Fig Validation of fixed-CpG binning approach. (A) Histogram of bin lengths for the comparison in Fig 2B using bins covering 50, 20 or 10 CpGs. For illustrative purpose, the first histograms were calculated for > 90% of all bins. Mean and median bin lengths are indicated by black and green lines, respectively. (B) Number of DMRs (p<0.05, ±10%< DNA methylation difference) between each longevity intervention and it’s respective control using bins covering 50, 20 or 10 CpGs. Proportion of hypermethylated DMRs are indicated in %. (C) Scatterplot representation of methylation values of each 10-CpG bin in control and long-lived animals. Intervention-related DNA methylation changes are highlighted. Bins that were not differentially regulated (background) are represented in grey. Data density is indicated by color code.
(TIF)

S3 Fig Conservation of hypermethylation patterns is independent of window size. (A) Venn diagram depicting the overlap of significantly hypermethylated DMRs under DR, Dwarfism and rapamycin treatment relative to the control groups at old age using bins covering 50, 20 or 10 CpGs (p-values; *** p<0.001, ** p<0.01, * p<0.05, *Fisher’s exact* test). (B) Enrichment analysis of common hypermethylated 10-CpG-DMRs over common hypermethylated DMRs detected with 50-CpG bins. Bars indicate the ratio of the observed DMR frequency and the average frequency across the genome (log2-transformed; adjusted p-values; *** p<0.001, ** p<0.01, * p<0.05, *Fisher’s exact* test). (C) Venn diagram depicting the overlap of significantly hypomethylated DMRs under DR, Dwarfism and rapamycin treatment relative to the control groups at old age using bins covering 50, 20 or 10 CpGs (p-values; *** p<0.001, ** p<0.01, * p<0.05, *Fisher’s exact* test).
(TIF)

S4 Fig Enrichment analysis of intervention-related DMRs with increased resolution. (A-C) Enrichment analysis of common hypermethylated 10-CpG-DMRs between DR and Dwarfs (n = 6679) and rapamycin treatment and Ames dwarf mice (n = 4355) over genomic elements (A), ENCODE chromatin states (B) and Cistrome binding sites of DNA binding elements (C). Bars indicate the ratio of the observed DMR frequency and the average frequency across the genome (log2-transformed; adjusted p-values; *** p<0.001, ** p<0.01, * p<0.05, *Fisher’s exact* test). (D) Differential methylation landscape of the Elovl6, Egf, Ghr, and Egfr gene loci using 10-CpG bins. Bins are represented as bars with color scale and height indicating methylation differences. Shaded area indicates location of 50-CpG-DMRs for comparison. Arrows indicate gene orientation; merged mRNA structure is depicted below.
(TIF)

S5 Fig Functional enrichment analysis of intervention-related DMRs. Functional enrichment of differentially hyper- and hypomethylated genes in DR-treated mice (A), rapamycin-treated mice (B) and Ames dwarf mice (C) at old age in comparison to respective controls.
(TIF)

S6 Fig Cross-strain hepatic DNA methylation changes in response to ageing. (A) Schematic representation of data sets and comparisons made. We analyzed age-related DMRs, comparing old and young control mice for each strain separately. Pre-processing was conducted in parallel for all groups (B) Boxplot representation of global DNA methylation levels in the liver of young and old C3B6F1♀, heterozygous Prop1 mutant and UM-HET3♀ mice. Strain and sex are indicated above. (C) Number of significantly differentially methylated regions (age-related DMRs) (p<0.05, ±10%< DNA methylation difference) in response to age in three different mouse strains. Proportion of hypermethylated DMRs are indicated in %. (D) Scatterplot representation of methylation values of each bin in young and old animals compared across strains. Age-related DNA methylation changes are highlighted. Bins that were not differentially regulated (background) are represented in grey. Data density is indicated by color code. (E,F) Venn diagram depicting the overlap of significantly age-related hypo- (E) and hypermethylated (F) DMRs across mouse strains. The set of age-related DMRs detected in at least two strains are highlighted in grey (p-values; *** p<0.001, ** p<0.01, * p<0.05, *Fisher’s exact* test).
(TIF)

S7 Fig Longevity intervention-related differential methylation is initiated at young age and does not result from retardation of ageing alone. (A) Venn diagram depicting the overlap of bins located over hypomethylated age-related DMRs with hypermethylated DMRs common between DR, dwarfism and rapamycin treatment (p-values; *** p<0.001, ** p<0.01, * p<0.05, *Fisher’s exact* test). (B) Schematic representation of data sets and comparisons. We probed for intervention-related DMRs at young age, by comparing long-lived mice to their respective controls. Pre-processing was conducted in parallel for all shown treatment groups. (C) Number of DMRs (p<0.05, ±10%< DNA methylation difference) between young DR or young Dwarf mice and their respective controls. Proportion of hypermethylated DMRs are indicated in %. (D) Scatterplot comparison of bin-wise differences between Young DR-related DMRs versus changes in young Dwarf mice, respectively. Bins overlapping the Scd1 gene are highlighted in red. Methylation differences under DR were significantly positively associated with differences in Ames dwarf mice (One-sided *Fisher’s exact* test p < 0.01). Number of bins in each quadrant is indicated in blue. (E) Enrichment analysis of hypermethylated DMRs under young DR or young Dwarf mice over longevity-related DMRs detected at old age. Bars indicate the ratio of the observed DMR frequency and the average frequency across the genome (log2-transformed; adjusted p-values; *** p<0.001, ** p<0.01, * p<0.05, *Fisher’s exact* test). (F,G) Venn diagram depicting the overlap of hypermethylated genes (≥ 2 DMRs overlapping) under young DR, young Ames dwarf and (F) old DR or (G) old Ames dwarf mice, respectively (p-values; *** p<0.001, ** p<0.01, * p<0.05, *Fisher’s exact* test). (H,I) Differential methylation landscape of the Scd1 (H) and Elovl6 (I) gene loci in DR or Ames dwarf mice at young age. Bins are represented as bars with color scale and height indicating methylation differences. Arrows indicate gene orientation; merged mRNA structure is depicted below.
(TIF)

S8 Fig Concerted CpG hypermethylation in four distinct DMRs occurs in response to DR, reduced somtaotropic signaling and rapamycin-treatment. (A) Pictographic representations of chromosome 8 with intervention-related DMRs indicated by colored bars. Highlighted is a DMR common among all three models that was not detected as age-related DMR. (B) The shaded area indicates the hypermethylated DMR common among all three longevity models mapping over the Tat gene locus. For improved resolution, the methylation profile is represented by 500 bp bins overlapping adjacent bins by 400 bp. Arrows indicate gene orientation; merged mRNA structure is depicted below. (C) DNA methylation levels of single cytosines (points) present in four longevity-related, age-independent DMRs in the liver of aged DR (C3B6F1♀ and UM-HET3♀), Ames Dwarf and Rapamycin treated mice next to their respective controls. Lines represent the methylation change over individual cytosines. Colored boxplots represent methylation levels averaged across all cytosines (p-values; *** p<0.001, ** p<0.01, * p<0.05, Paired *Wilcoxon rank-sum* test).
(TIF)

S9 Fig Methylation levels at 125 cytosines are correlated with longevity. (A) DNA methylation levels of single cytosines (points) present in four longevity intervention-related, age-independent 500 bp regions in the liver of aged DR (C3B6F1♀ and UM-HET3♀), Ames Dwarf and rapamycin-treated mice next to their respective controls. Lines represent the methylation change over individual cytosines. Colored boxplots represent methylation levels averaged across all cytosines (p-values; *** p<0.001, ** p<0.01, * p<0.05, Paired *Wilcoxon rank-sum* test). (B) DNA methylation levels over the same set of cytosines in young and old controls (p-values; *** p<0.001, ** p<0.01, * p<0.05, Paired *Wilcoxon rank-sum* test). (C) DNA methylation levels over the same set of cytosines in young Ames dwarf and DR (C3B6F1♀) mice next to their respective controls (p-values; *** p<0.001, ** p<0.01, * p<0.05, Paired *Wilcoxon rank-sum* test). (D) DNA methylation levels over the same set of cytosines measured by RRBS-seq in blood of adult DR (B6D2F1♂) mice next to their respective controls. The dashed box indicates the range of methylation levels that is not covered by RRBS-seq data in blood.
(TIF)

S10 Fig Verification of longevity marker by Bisulfite Amplicon sequencing (BS-AS). (A) CpG-wise DNA methylation at the four targeted DMRs as measured by BS-AS in exemplary Irs1KO replicate. DNA methylation as measured by WGBS-seq (Dietary restriction) is displayed on top for comparison. Target DMRs and surrounding genes and CGIs are highlighted. Genomic location of the marker regions are listed in the table above (B) Heatmap of unsupervised clustering of CpG-wise methylation changes as measured by BS-AS (n = 4–5 replicates per group; color bar represents *z*-score range). Additionally, replicates of the same treatment group were averaged and provided as additional sample for comparison with individual replicates. Clustering of CpGs dependent on region (rows) and treatment group (columns) are highlighted. White box indicates Region III. (C,D) DNA methylation levels of single cytosines (points; n = 80) present in (C) four or (D) three longevity-related, age-independent DMRs in the liver of aged DR fed (C3B6F1♀) and Irs1-KO mice next to their respective controls. Lines represent the methylation change over individual cytosines. Red lines indicate dCpGs with higher methylation levels in long-lived mice; green lines indicate dCpGs with lower methylation levels. Colored boxplots represent methylation levels averaged across all cytosines (p-values; *** p<0.001, ** p<0.01, * p<0.05, Paired *Wilcoxon rank-sum* test).
(TIF)

S11 Fig Dwarfism-related DNA methylation differences predict expression changes in young animals. (A) Scatterplot of expression differences versus methylation differences of dwarfism-related DMRs at young age. Dashed lines indicate DNA methylation cutoff of > ±10%. DNA hypermethylation associated significantly with reduced gene expression (*Binomial* test p < 0.001). Number of differentially methylated genes in each quadrant is indicated in blue and red, for all genes and differentially expressed genes, respectively. (B) Gene ontology and Reactome enrichment of genes with a negative correlation of gene expression and methylation. Lengths of bars represent negative ln-transformed, adjusted pvalues using *Fisher’s exact* test. Cells indicate log2-foldchanges (log2FC) between Ames dwarf and controls per gene. (C) Venn diagram depicting the overlap of genes with a negative correlation of gene expression and methylation in young and old Ames Dwarf mice (p-values; *** p<0.001, ** p<0.01, * p<0.05, *Fisher’s exact* test). (D,E) Scatterplot of expression differences versus methylation differences of dwarfism-related DMRs mapping uiDMRs at (D) young and (E) old age. Dashed lines indicate DNA methylation cutoff of > ±10%. DNA hypermethylation associated significantly with reduced gene expression (*Binomial* test p < 0.001). Number of differentially methylated genes in each quadrant is indicated in blue and red, for all genes and differentially expressed genes, respectively.
(TIF)

S12 Fig DR and genetic dwarfism commonly regulate methylation and expression of Fgfr3. (A) Scatterplot of expression differences versus methylation differences of DR-related DMRs. Dashed lines indicate DNA methylation cutoff of > ±10%. DNA hypermethylation associated significantly with reduced gene expression (*Binomial* test p < 0.05). Number of differentially methylated genes in each quadrant is indicated in blue and red, for all genes and differentially expressed genes, respectively. (B) Gene ontology and Reactome enrichment of genes with a negative correlation of gene expression and methylation. Lengths of bars represent negative ln-transformed, adjusted pvalues using *Fisher’s exact* test. Cells indicate log2-foldchanges (log2FC) between DR mice and control group per gene. (C,D) Venn diagrams depicting the overlap of genes being transcriptionally (C) down- or (D) up-regulated in DR or Ames dwarf mice, respectively. Both down- and up-regulated genes showed a significant overlap between both longevity models (p-values; *** p<0.001, ** p<0.01, * p<0.05, *Fisher’s exact* test). (E) Venn diagram depicting the overlap of genes being transcriptionally down-regulated and hypermethylated in DR and Ames dwarf mice. (F) Differential methylation landscape of the Fgfr3 gene locus in DR, Rapamycin treated and Ames dwarf mice. Bins are represented as bars with color scale and height indicating methylation differences. Arrows indicate gene orientation; merged mRNA structure is depicted below. (G) Fgfr3 mRNA expression by RNA-sequencing in control and Ames Dwarf mice at young and old age, respectively (n = 4 vs 2 and 4 vs 4). (H) Fgfr3 mRNA expression by RNA-sequencing in control and DR mice at young and old age, respectively (n = 3 vs 3 and 3 vs 3).
(TIF)

S13 Fig *In-vitro* knockdown of Fgfr3 regulates lipid metabolism similar to DR *in-vivo*. (A) Venn diagram depicting the overlap of genes differentially expressed in livers of DR treated mice and RT112 cells transfected with short-hairpin RNA targeting Fgfr3 (p-values; *** p<0.001, ** p<0.01, * p<0.05, *Fisher’s exact* test). (B) Scatterplot of expression differences in livers of DR treated mice versus expression differences in RT112 cells with induced Fgfr3 knockdown. Number of differentially expressed genes in each quadrant is indicated in blue with common expression changes highlighted in red. Both transcriptomes showed significantly common expression signatures (p < 0.01, *Fisher’s exact* test). (C) Gene ontology and Reactome enrichment of genes being commonly regulated in DR treated mice and RT112 cells with induced Fgfr3 knockdown. Lengths of bars represent negative ln-transformed, adjusted pvalues using *Fisher’s exact* test. Cells indicate log2-foldchanges (log2FC) between DR mice and control group per gene.
(TIF)

S1 Table Overview of datasets used in this study. (XLSX)

S2 Table List of DR-related DMRs in two strains. (XLSX)

S3 Table Functional enrichment of genes associated with identical DR-related DMRs in two strains. (XLSX)

S4 Table Functional enrichment of genes associated with DR-related hypermethylation in two strains. (XLSX)

S5 Table List of DR-, rapamycin-, and dwarfism-related DMRs at old age. (XLSX)

S6 Table Functional enrichment of genes associated with differential methylation in female DR fed C3HB6F1 mice. (XLSX)

S7 Table Functional enrichment of genes associated with differential methylation in female rapamycin-treated UM-HET3 mice. (XLSX)

S8 Table Functional enrichment of genes associated with differential methylation in male Ames dwarf mice. (XLSX)

S9 Table List of genes associated with hypermethylation in DR, rapamycin-treated or Ames dwarf mice at old age. (XLSX)

S10 Table Functional enrichment of genes associated with hypermethylation under DR feeding and in Ames dwarf mice. (XLSX)

S11 Table Functional enrichment of genes associated with hypermethylation in rapamycin-treated and in Ames dwarf mice. (XLSX)

S12 Table List of age-related DMRs in three mouse strains. (XLSX)

S13 Table List of DR- and dwarfism-related DMRs at young age. (XLSX)

S14 Table Location and methylation levels of 125 putative marker cytosines. (XLSX)

S15 Table Methylation and expression comparison of genes associated with dwarfism-related DMRs at old age. (XLSX)

S16 Table Functional enrichment of hypermethylated and down-regulated genes in old Ames dwarf mice. (XLSX)

S17 Table Methylation and expression comparison of genes associated with dwarfism-related DMRs at young age. (XLSX)

S18 Table Functional enrichment of hypermethylated and down-regulated genes in young Ames dwarf mice. (XLSX)

S19 Table Methylation and expression comparison of genes associated with DR-related DMRs at old age. (XLSX)

S20 Table Functional enrichment of hypermethylated and down-regulated genes in old DR fed C3HB6F1 mice. (XLSX)

S21 Table Comparison of differentially expressed genes under DR in-vivo and in-vitro knockdown of Fgfr3. (XLSX)

S22 Table Functional enrichment of genes showing consistent differential expression in DR fed C3HB6F1 mice and RT112 cells with induced Fgfr3 knockdown. (XLSX)

S23 Table List of barcoded primers used for BS-AS. (XLSX)
