# Supplementary figures and images for: Hepatic gene body hypermethylation is a shared epigenetic signature of murine longevity
Source: PLoS Genet. 2018 Nov 21;14(11):e1007766. doi: 10.1371/journal.pgen.1007766 (PMC6281273; doi:10.1371/journal.pgen.1007766)

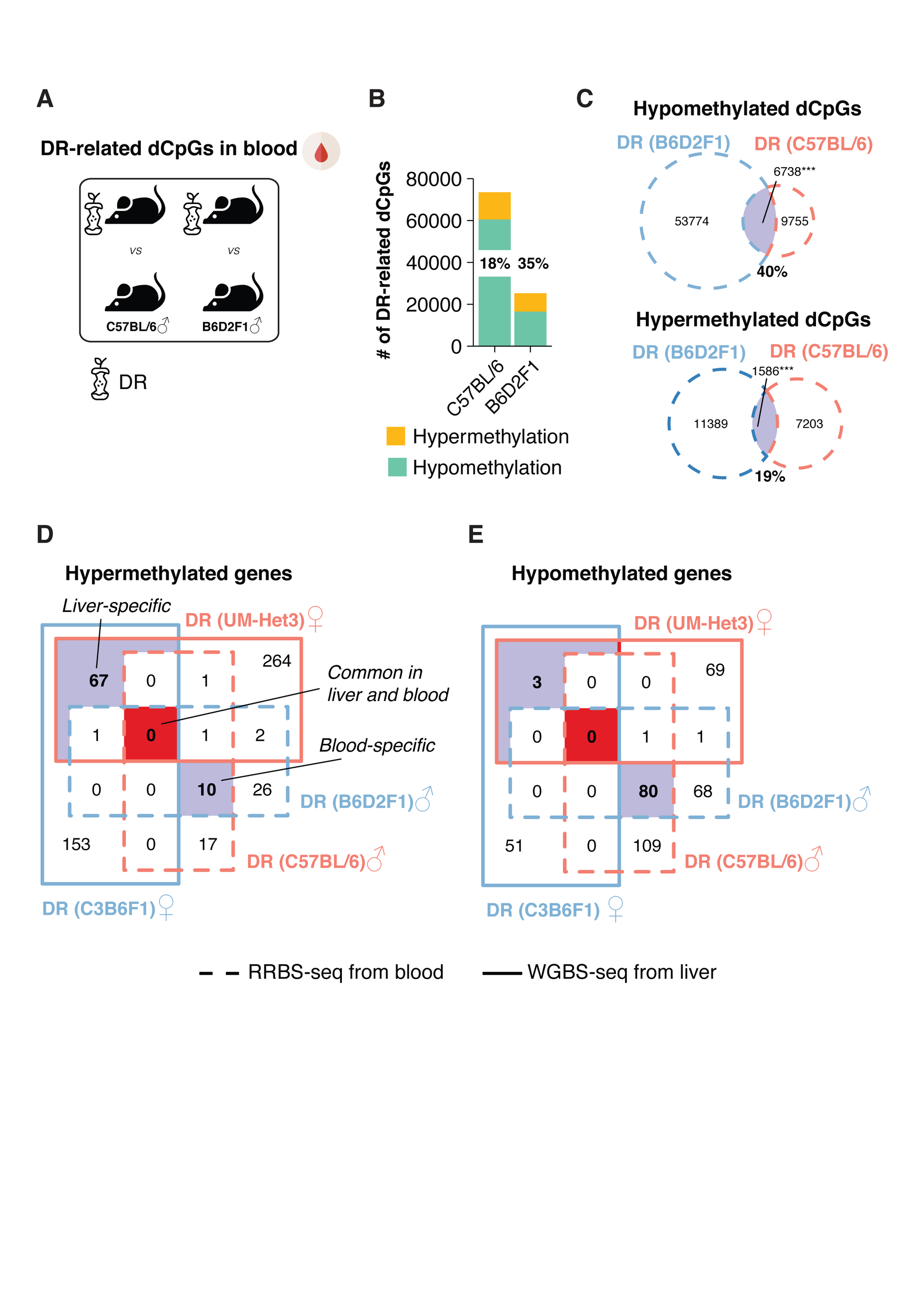

Supplement: S1 Fig — (A) Schematic representation of the data sets analysed and comparisons made. We probed for Dietary Restricted (DR)-related dCpGs in blood of adult B6D2F1♂ and C57BL/6♂. Pre-processing was conducted in parallel for all experimental groups (B) Number of dCpGs (p<0.05, ±10%< DNA methylation difference) between each DR cohort and its respective control. Proportion of hypermethylated dCpGs are indicated in %. (C) Venn diagrams depicting the overlap of significantly hypo- and hypermethylated dCpGs in both DR cohorts. Proportions of overlap relative to the C57BL/6♂ set are indicated in % (p-values; *** p<0.001, ** p<0.01, * p<0.05, Fisher’s exact test). (D,E) Edwards-Venn diagrams depicting the overlap of significantly hypo- (E) and hypermethylated (D) genes in liver (see Fig 1) and blood present in the four tested DR cohorts. Liver and blood datasets are indicated by solid and dashed lines, respectively. (TIF) [file pgen.1007766.s001.tif]

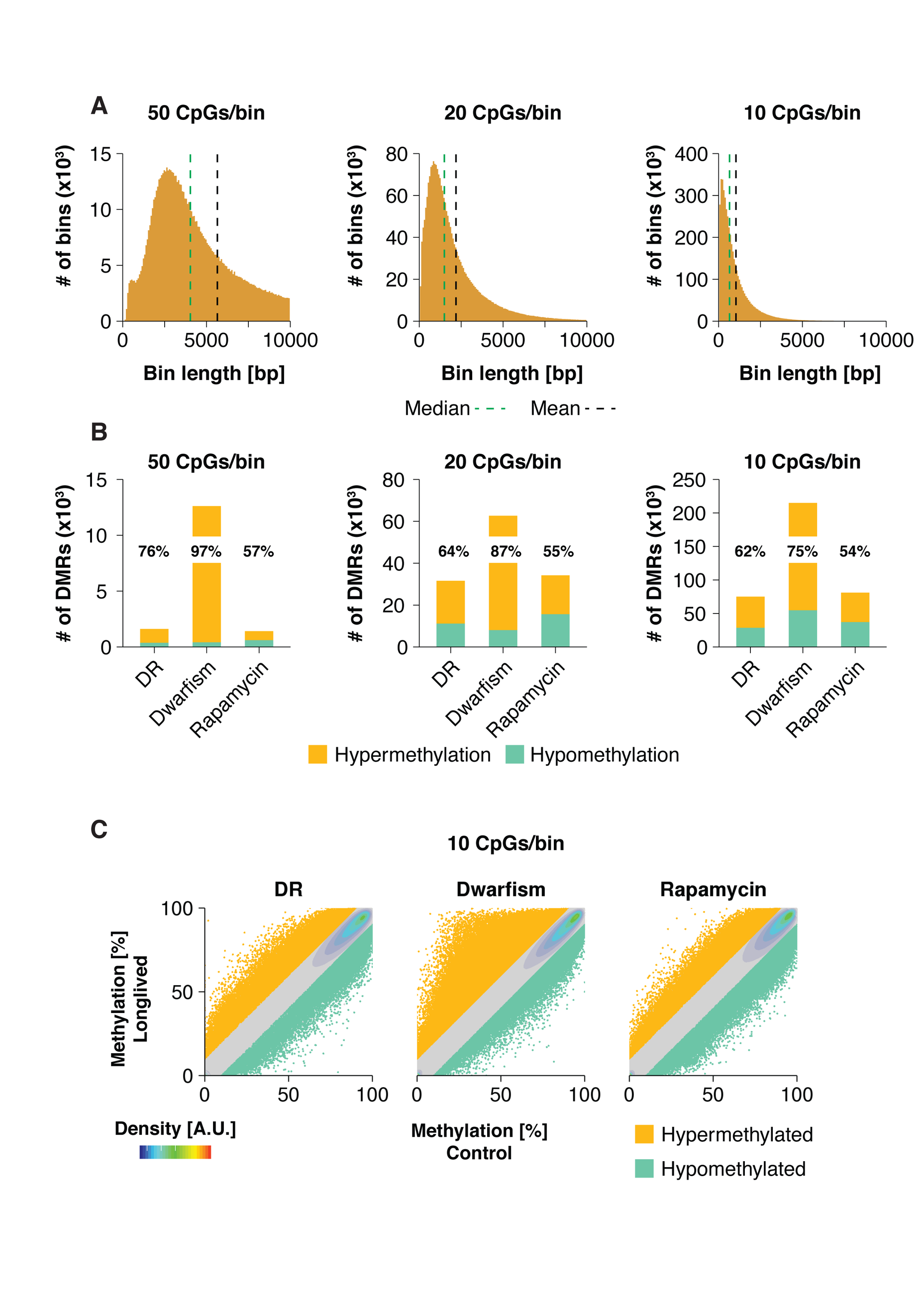

Supplement: S2 Fig — (A) Histogram of bin lengths for the comparison in Fig 2B using bins covering 50, 20 or 10 CpGs. For illustrative purpose, the first histograms were calculated for > 90% of all bins. Mean and median bin lengths are indicated by black and green lines, respectively. (B) Number of DMRs (p<0.05, ±10%< DNA methylation difference) between each longevity intervention and it’s respective control using bins covering 50, 20 or 10 CpGs. Proportion of hypermethylated DMRs are indicated in %. (C) Scatterplot representation of methylation values of each 10-CpG bin in control and long-lived animals. Intervention-related DNA methylation changes are highlighted. Bins that were not differentially regulated (background) are represented in grey. Data density is indicated by color code. (TIF) [file pgen.1007766.s002.tif]

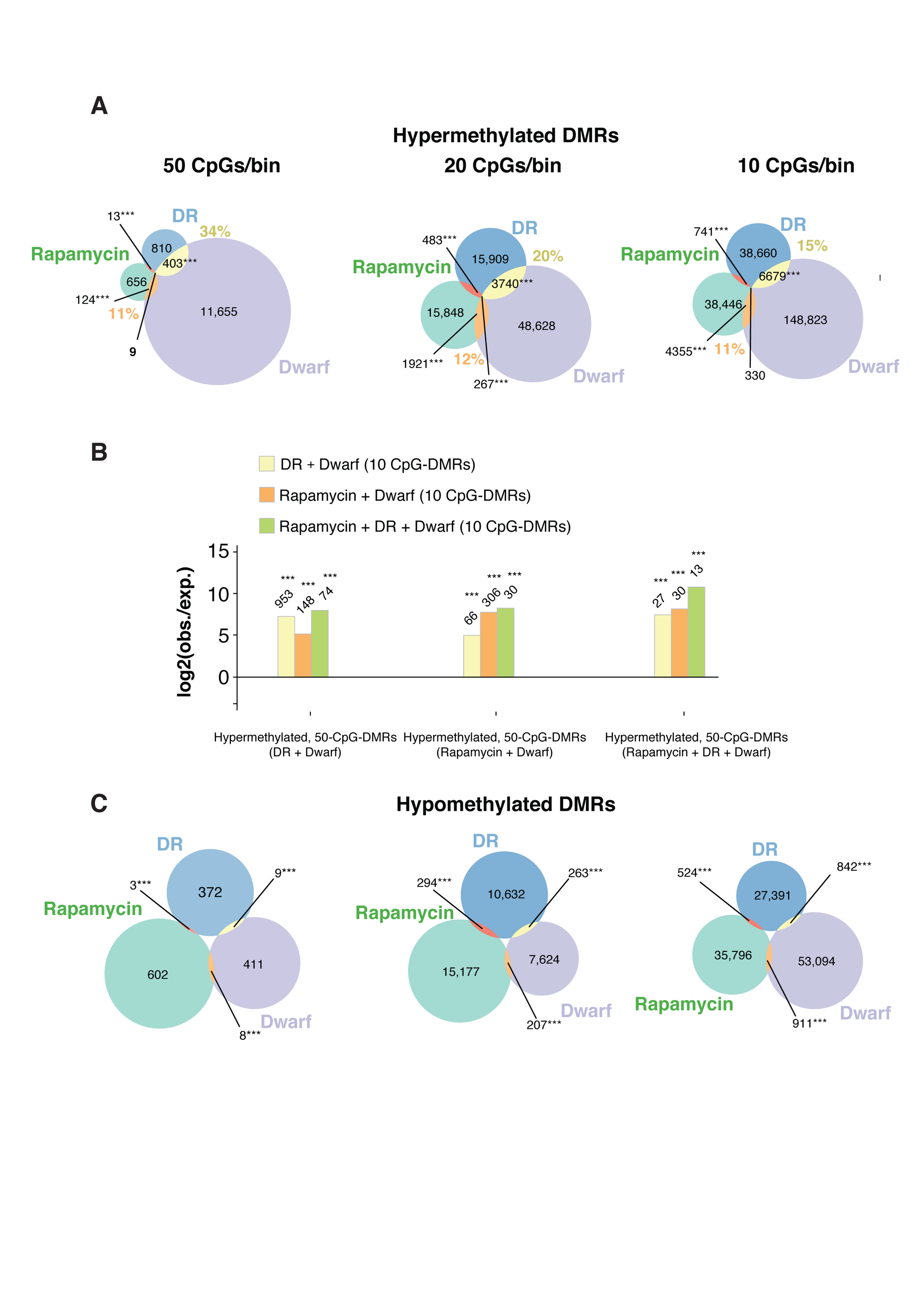

Supplement: S3 Fig — (A) Venn diagram depicting the overlap of significantly hypermethylated DMRs under DR, Dwarfism and rapamycin treatment relative to the control groups at old age using bins covering 50, 20 or 10 CpGs (p-values; *** p<0.001, ** p<0.01, * p<0.05, Fisher’s exact test). (B) Enrichment analysis of common hypermethylated 10-CpG-DMRs over common hypermethylated DMRs detected with 50-CpG bins. Bars indicate the ratio of the observed DMR frequency and the average frequency across the genome (log2-transformed; adjusted p-values; *** p<0.001, ** p<0.01, * p<0.05, Fisher’s exact test). (C) Venn diagram depicting the overlap of significantly hypomethylated DMRs under DR, Dwarfism and rapamycin treatment relative to the control groups at old age using bins covering 50, 20 or 10 CpGs (p-values; *** p<0.001, ** p<0.01, * p<0.05, Fisher’s exact test). (TIF) [file pgen.1007766.s003.tif]

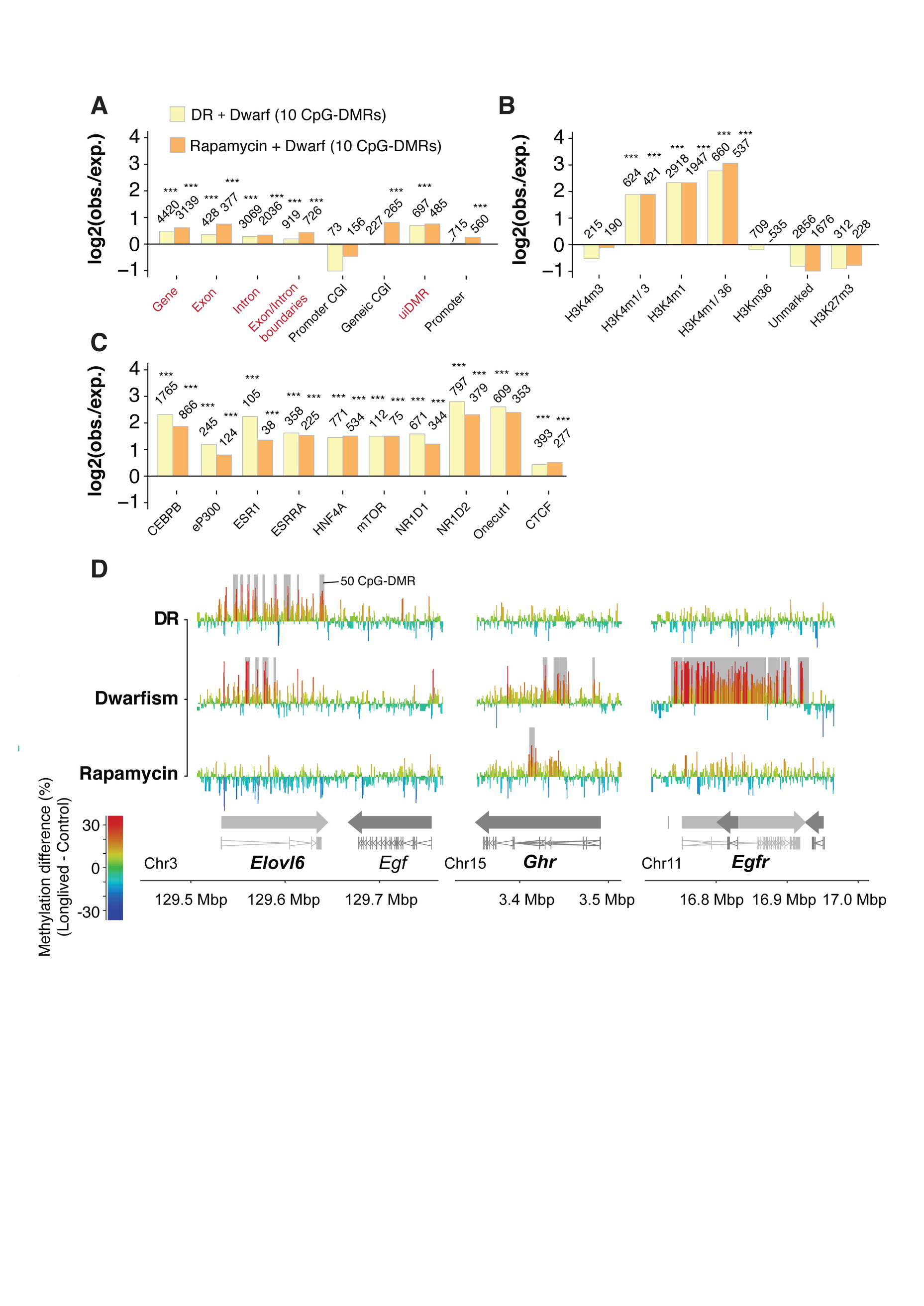

Supplement: S4 Fig — (A-C) Enrichment analysis of common hypermethylated 10-CpG-DMRs between DR and Dwarfs (n = 6679) and rapamycin treatment and Ames dwarf mice (n = 4355) over genomic elements (A), ENCODE chromatin states (B) and Cistrome binding sites of DNA binding elements (C). Bars indicate the ratio of the observed DMR frequency and the average frequency across the genome (log2-transformed; adjusted p-values; *** p<0.001, ** p<0.01, * p<0.05, Fisher’s exact test). (D) Differential methylation landscape of the Elovl6, Egf, Ghr, and Egfr gene loci using 10-CpG bins. Bins are represented as bars with color scale and height indicating methylation differences. Shaded area indicates location of 50-CpG-DMRs for comparison. Arrows indicate gene orientation; merged mRNA structure is depicted below. (TIF) [file pgen.1007766.s004.tif]

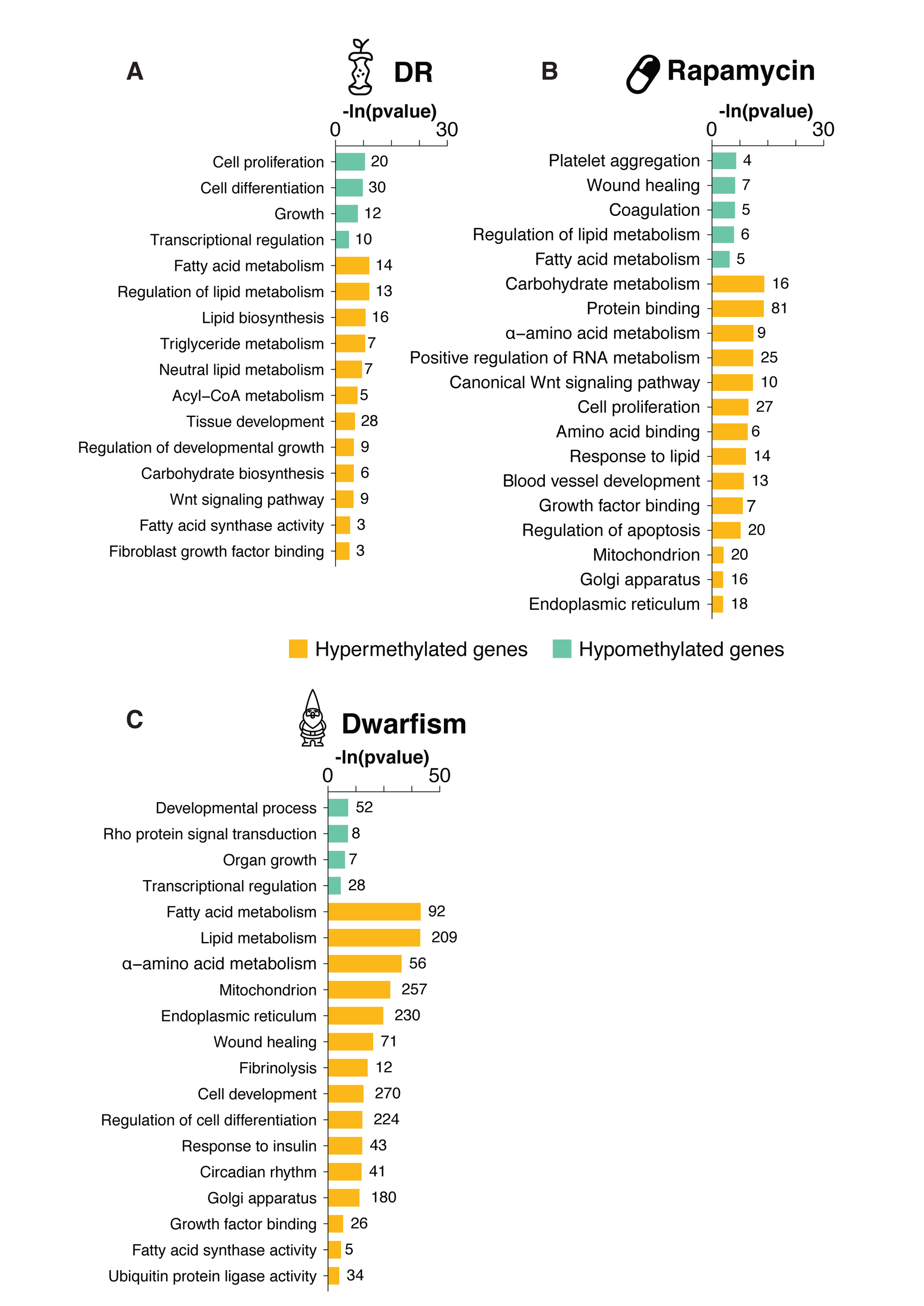

Supplement: S5 Fig — Functional enrichment of differentially hyper- and hypomethylated genes in DR-treated mice (A), rapamycin-treated mice (B) and Ames dwarf mice (C) at old age in comparison to respective controls. (TIF) [file pgen.1007766.s005.tif]

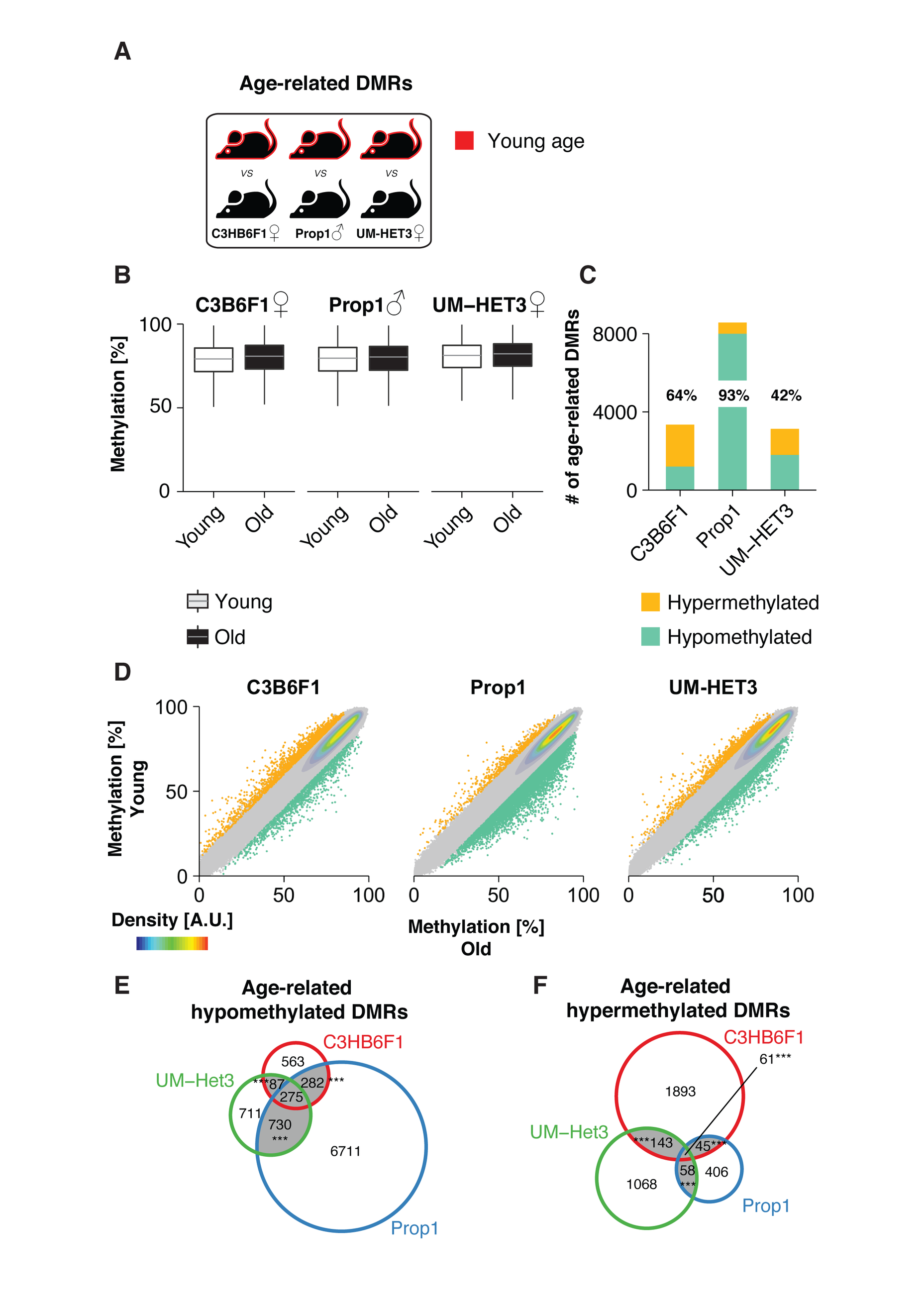

Supplement: S6 Fig — (A) Schematic representation of data sets and comparisons made. We analyzed age-related DMRs, comparing old and young control mice for each strain separately. Pre-processing was conducted in parallel for all groups (B) Boxplot representation of global DNA methylation levels in the liver of young and old C3B6F1♀, heterozygous Prop1 mutant and UM-HET3♀ mice. Strain and sex are indicated above. (C) Number of significantly differentially methylated regions (age-related DMRs) (p<0.05, ±10%< DNA methylation difference) in response to age in three different mouse strains. Proportion of hypermethylated DMRs are indicated in %. (D) Scatterplot representation of methylation values of each bin in young and old animals compared across strains. Age-related DNA methylation changes are highlighted. Bins that were not differentially regulated (background) are represented in grey. Data density is indicated by color code. (E,F) Venn diagram depicting the overlap of significantly age-related hypo- (E) and hypermethylated (F) DMRs across mouse strains. The set of age-related DMRs detected in at least two strains are highlighted in grey (p-values; *** p<0.001, ** p<0.01, * p<0.05, Fisher’s exact test). (TIF) [file pgen.1007766.s006.tif]

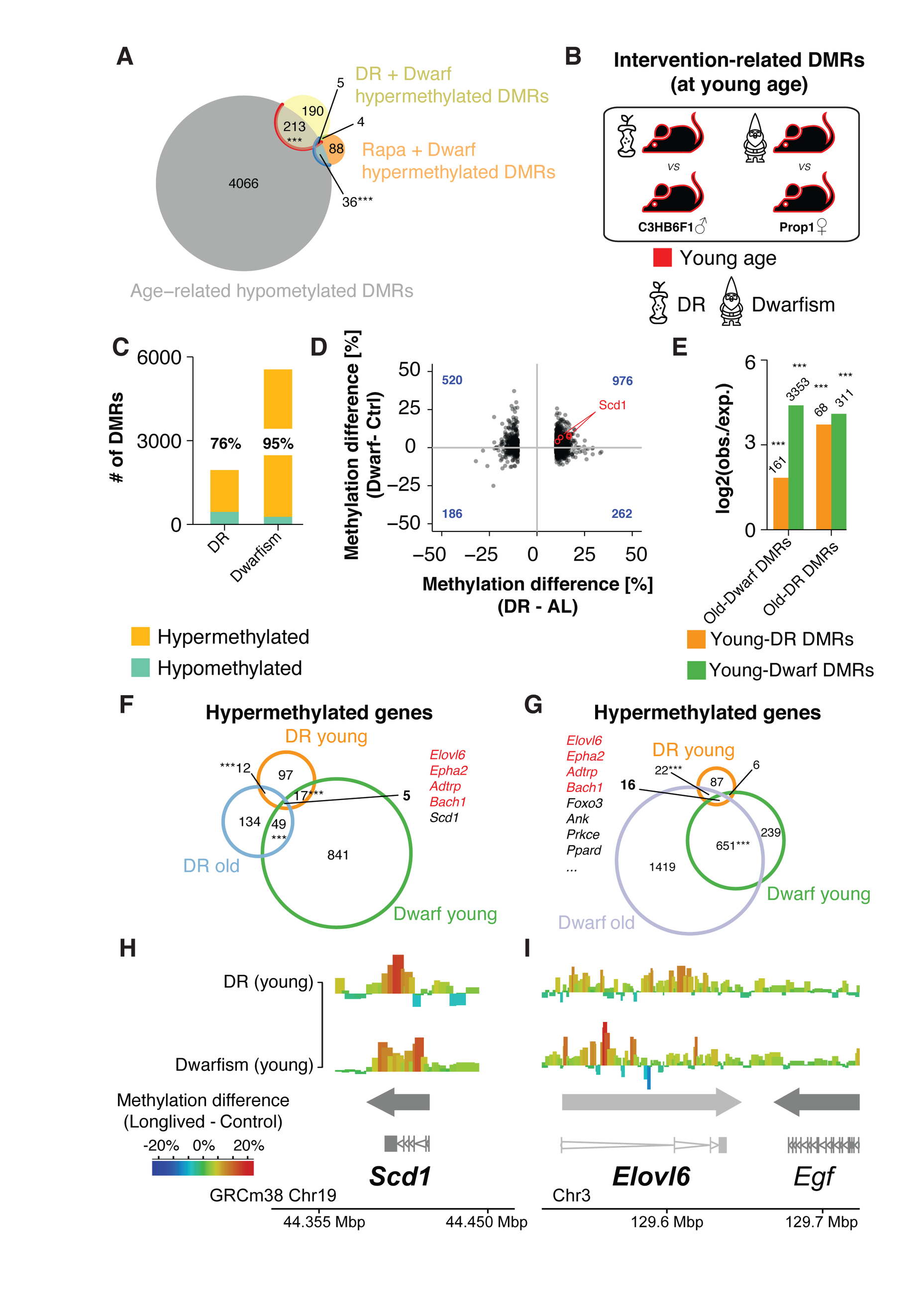

Supplement: S7 Fig — (A) Venn diagram depicting the overlap of bins located over hypomethylated age-related DMRs with hypermethylated DMRs common between DR, dwarfism and rapamycin treatment (p-values; *** p<0.001, ** p<0.01, * p<0.05, Fisher’s exact test). (B) Schematic representation of data sets and comparisons. We probed for intervention-related DMRs at young age, by comparing long-lived mice to their respective controls. Pre-processing was conducted in parallel for all shown treatment groups. (C) Number of DMRs (p<0.05, ±10%< DNA methylation difference) between young DR or young Dwarf mice and their respective controls. Proportion of hypermethylated DMRs are indicated in %. (D) Scatterplot comparison of bin-wise differences between Young DR-related DMRs versus changes in young Dwarf mice, respectively. Bins overlapping the Scd1 gene are highlighted in red. Methylation differences under DR were significantly positively associated with differences in Ames dwarf mice (One-sided Fisher’s exact test p < 0.01). Number of bins in each quadrant is indicated in blue. (E) Enrichment analysis of hypermethylated DMRs under young DR or young Dwarf mice over longevity-related DMRs detected at old age. Bars indicate the ratio of the observed DMR frequency and the average frequency across the genome (log2-transformed; adjusted p-values; *** p<0.001, ** p<0.01, * p<0.05, Fisher’s exact test). (F,G) Venn diagram depicting the overlap of hypermethylated genes (≥ 2 DMRs overlapping) under young DR, young Ames dwarf and (F) old DR or (G) old Ames dwarf mice, respectively (p-values; *** p<0.001, ** p<0.01, * p<0.05, Fisher’s exact test). (H,I) Differential methylation landscape of the Scd1 (H) and Elovl6 (I) gene loci in DR or Ames dwarf mice at young age. Bins are represented as bars with color scale and height indicating methylation differences. Arrows indicate gene orientation; merged mRNA structure is depicted below. (TIF) [file pgen.1007766.s007.tif]

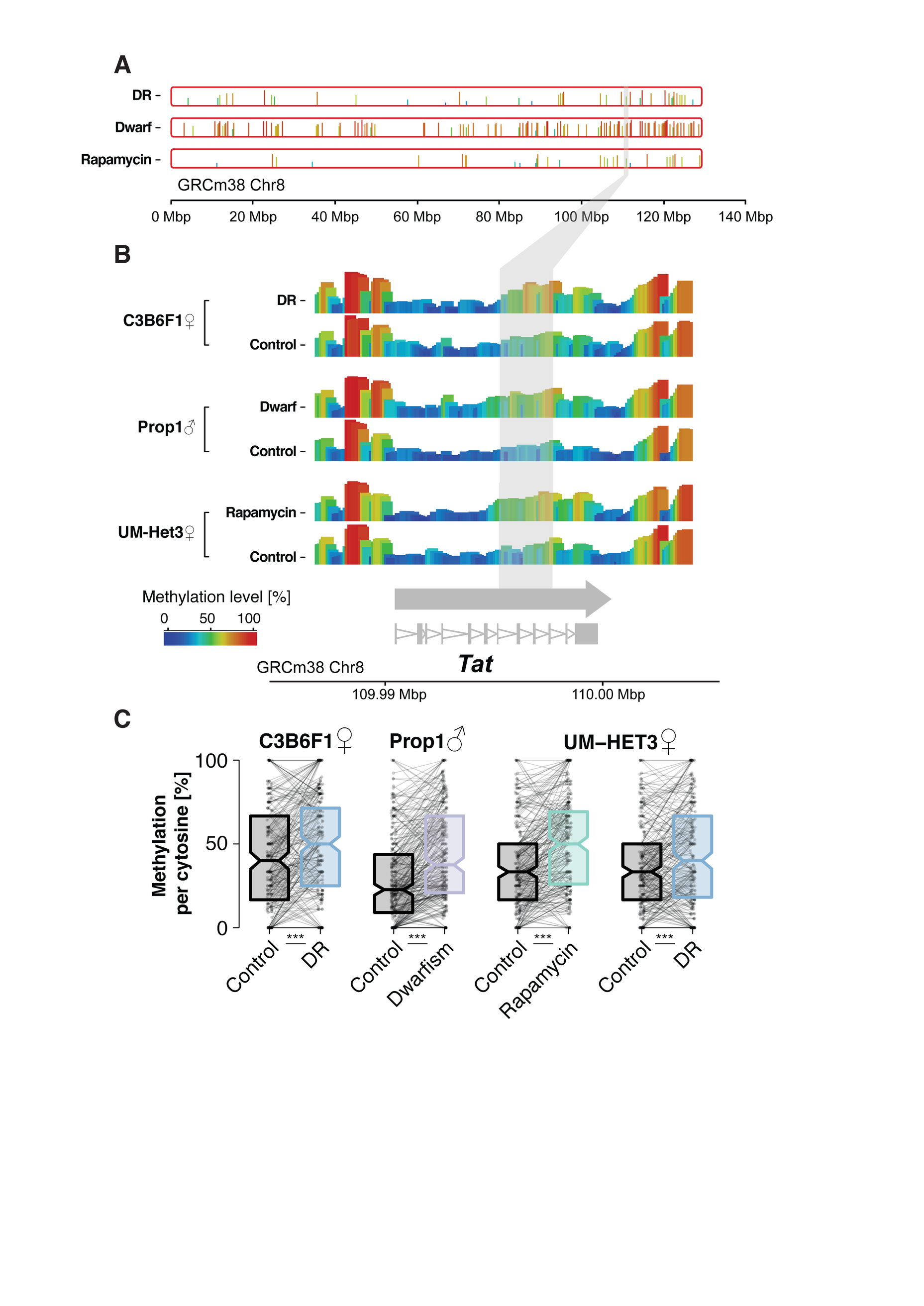

Supplement: S8 Fig — (A) Pictographic representations of chromosome 8 with intervention-related DMRs indicated by colored bars. Highlighted is a DMR common among all three models that was not detected as age-related DMR. (B) The shaded area indicates the hypermethylated DMR common among all three longevity models mapping over the Tat gene locus. For improved resolution, the methylation profile is represented by 500 bp bins overlapping adjacent bins by 400 bp. Arrows indicate gene orientation; merged mRNA structure is depicted below. (C) DNA methylation levels of single cytosines (points) present in four longevity-related, age-independent DMRs in the liver of aged DR (C3B6F1♀ and UM-HET3♀), Ames Dwarf and Rapamycin treated mice next to their respective controls. Lines represent the methylation change over individual cytosines. Colored boxplots represent methylation levels averaged across all cytosines (p-values; *** p<0.001, ** p<0.01, * p<0.05, Paired Wilcoxon rank-sum test). (TIF) [file pgen.1007766.s008.tif]

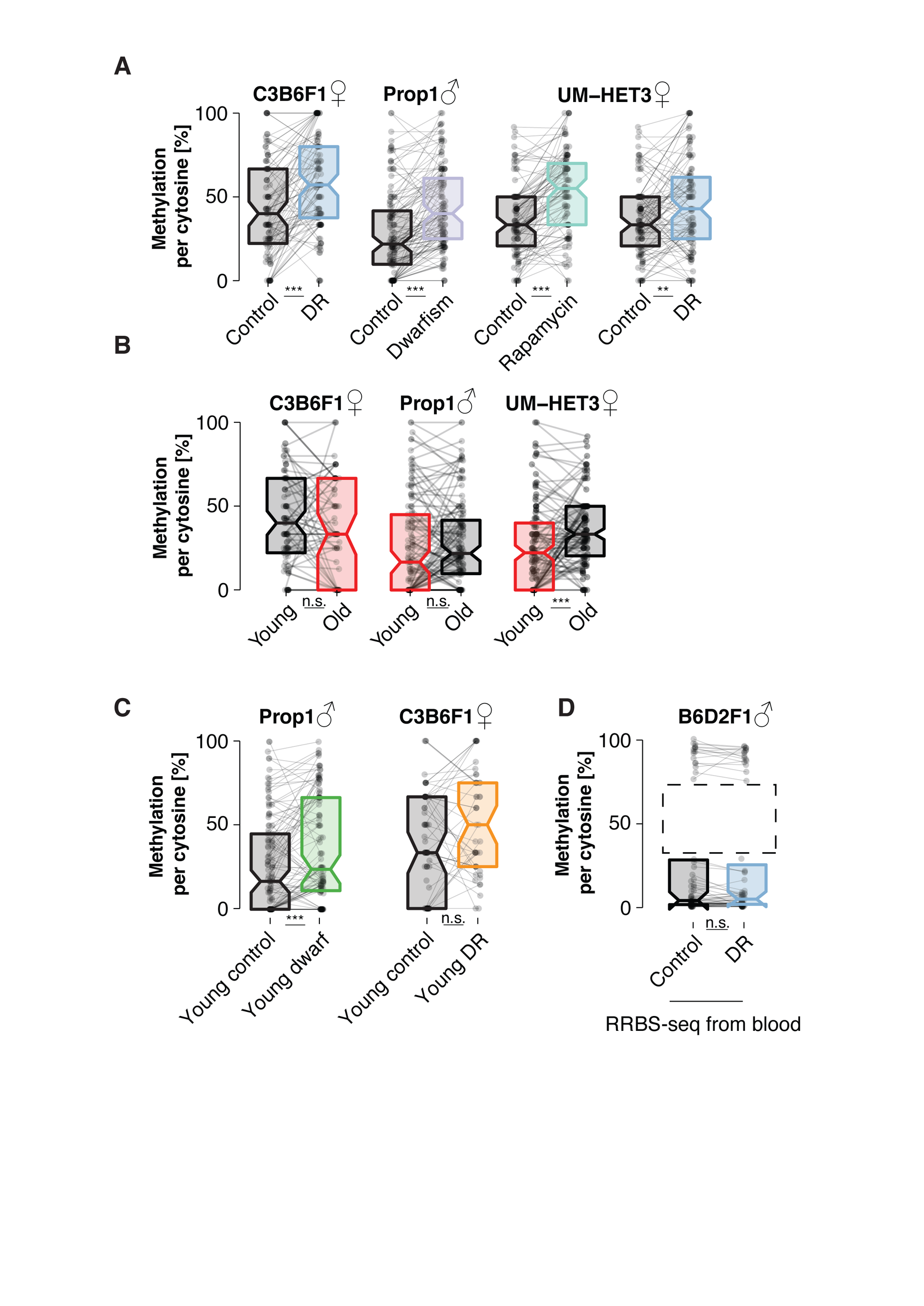

Supplement: S9 Fig — (A) DNA methylation levels of single cytosines (points) present in four longevity intervention-related, age-independent 500 bp regions in the liver of aged DR (C3B6F1♀ and UM-HET3♀), Ames Dwarf and rapamycin-treated mice next to their respective controls. Lines represent the methylation change over individual cytosines. Colored boxplots represent methylation levels averaged across all cytosines (p-values; *** p<0.001, ** p<0.01, * p<0.05, Paired Wilcoxon rank-sum test). (B) DNA methylation levels over the same set of cytosines in young and old controls (p-values; *** p<0.001, ** p<0.01, * p<0.05, Paired Wilcoxon rank-sum test). (C) DNA methylation levels over the same set of cytosines in young Ames dwarf and DR (C3B6F1♀) mice next to their respective controls (p-values; *** p<0.001, ** p<0.01, * p<0.05, Paired Wilcoxon rank-sum test). (D) DNA methylation levels over the same set of cytosines measured by RRBS-seq in blood of adult DR (B6D2F1♂) mice next to their respective controls. The dashed box indicates the range of methylation levels that is not covered by RRBS-seq data in blood. (TIF) [file pgen.1007766.s009.tif]

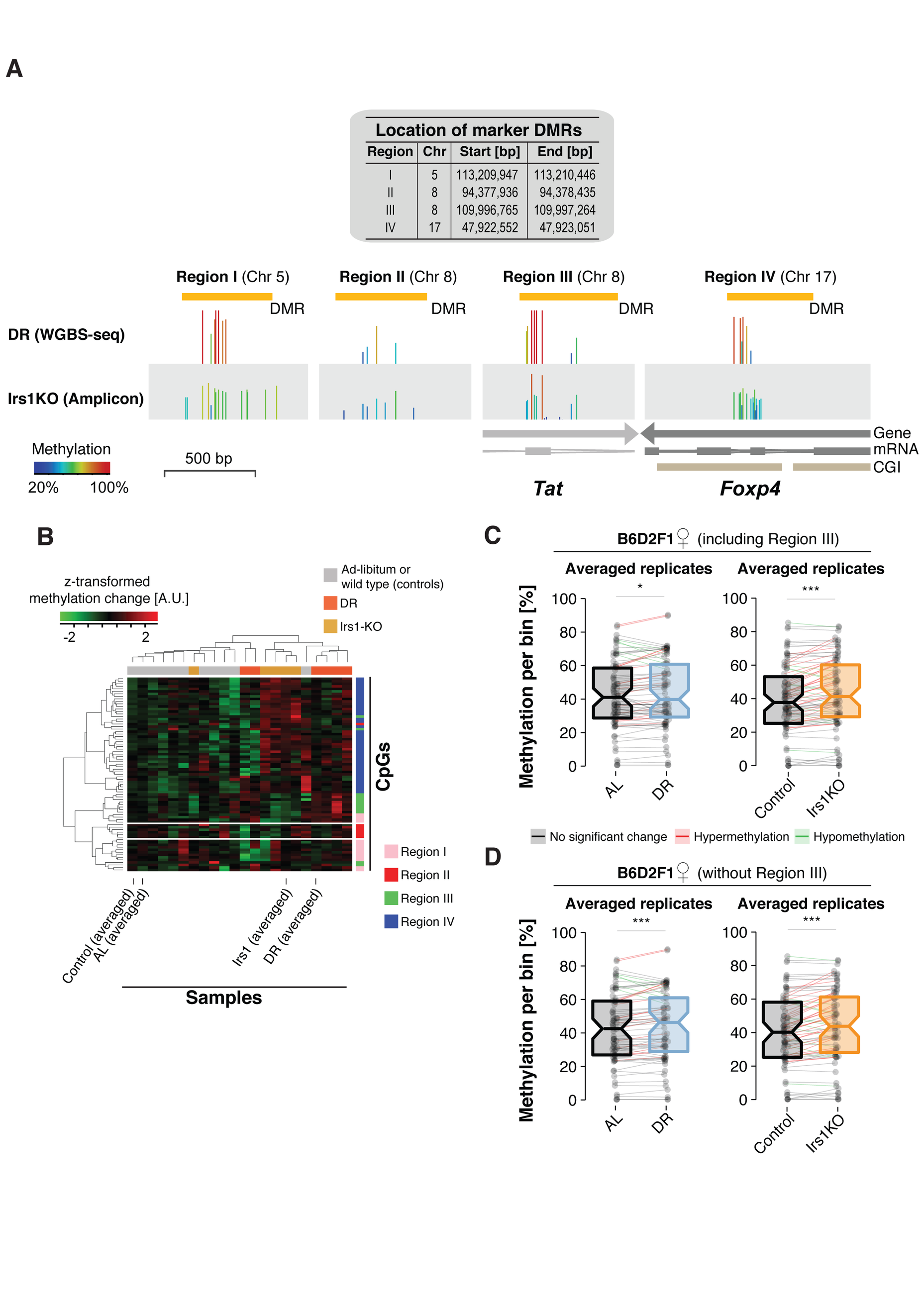

Supplement: S10 Fig — (A) CpG-wise DNA methylation at the four targeted DMRs as measured by BS-AS in exemplary Irs1KO replicate. DNA methylation as measured by WGBS-seq (Dietary restriction) is displayed on top for comparison. Target DMRs and surrounding genes and CGIs are highlighted. Genomic location of the marker regions are listed in the table above (B) Heatmap of unsupervised clustering of CpG-wise methylation changes as measured by BS-AS (n = 4–5 replicates per group; color bar represents z-score range). Additionally, replicates of the same treatment group were averaged and provided as additional sample for comparison with individual replicates. Clustering of CpGs dependent on region (rows) and treatment group (columns) are highlighted. White box indicates Region III. (C,D) DNA methylation levels of single cytosines (points; n = 80) present in (C) four or (D) three longevity-related, age-independent DMRs in the liver of aged DR fed (C3B6F1♀) and Irs1-KO mice next to their respective controls. Lines represent the methylation change over individual cytosines. Red lines indicate dCpGs with higher methylation levels in long-lived mice; green lines indicate dCpGs with lower methylation levels. Colored boxplots represent methylation levels averaged across all cytosines (p-values; *** p<0.001, ** p<0.01, * p<0.05, Paired Wilcoxon rank-sum test). (TIF) [file pgen.1007766.s010.tif]

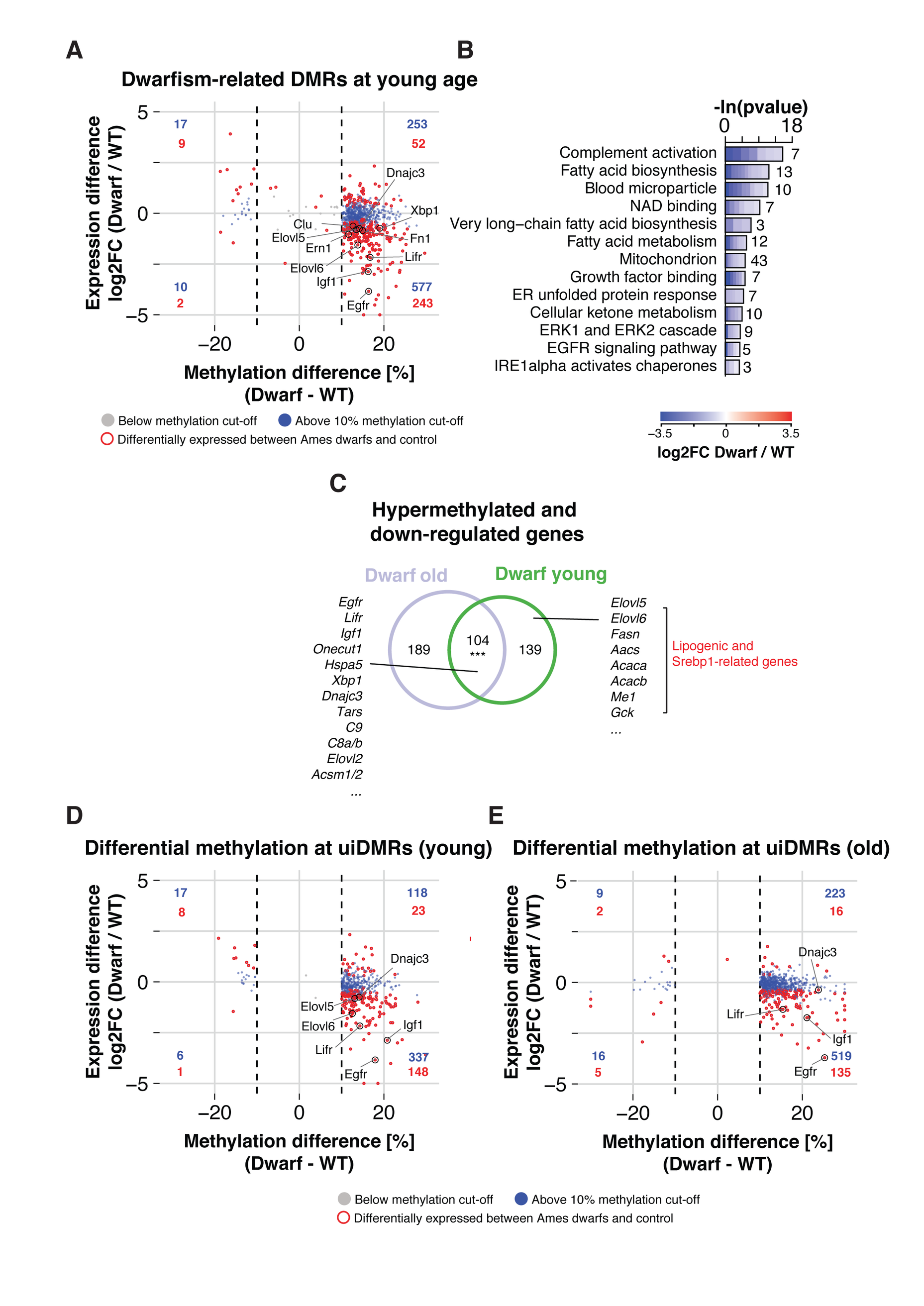

Supplement: S11 Fig — (A) Scatterplot of expression differences versus methylation differences of dwarfism-related DMRs at young age. Dashed lines indicate DNA methylation cutoff of > ±10%. DNA hypermethylation associated significantly with reduced gene expression (Binomial test p < 0.001). Number of differentially methylated genes in each quadrant is indicated in blue and red, for all genes and differentially expressed genes, respectively. (B) Gene ontology and Reactome enrichment of genes with a negative correlation of gene expression and methylation. Lengths of bars represent negative ln-transformed, adjusted pvalues using Fisher’s exact test. Cells indicate log2-foldchanges (log2FC) between Ames dwarf and controls per gene. (C) Venn diagram depicting the overlap of genes with a negative correlation of gene expression and methylation in young and old Ames Dwarf mice (p-values; *** p<0.001, ** p<0.01, * p<0.05, Fisher’s exact test). (D,E) Scatterplot of expression differences versus methylation differences of dwarfism-related DMRs mapping uiDMRs at (D) young and (E) old age. Dashed lines indicate DNA methylation cutoff of > ±10%. DNA hypermethylation associated significantly with reduced gene expression (Binomial test p < 0.001). Number of differentially methylated genes in each quadrant is indicated in blue and red, for all genes and differentially expressed genes, respectively. (TIF) [file pgen.1007766.s011.tif]

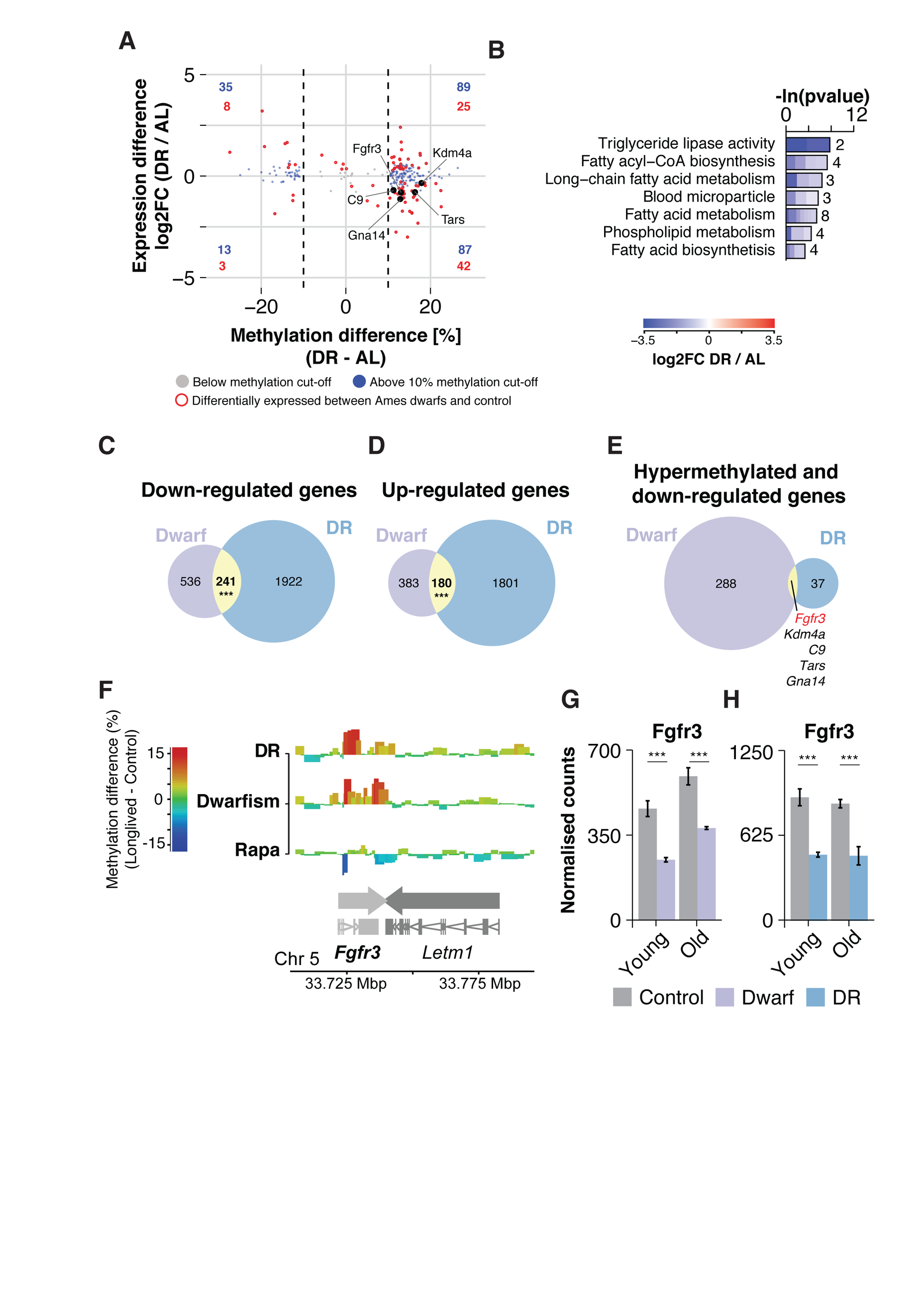

Supplement: S12 Fig — (A) Scatterplot of expression differences versus methylation differences of DR-related DMRs. Dashed lines indicate DNA methylation cutoff of > ±10%. DNA hypermethylation associated significantly with reduced gene expression (Binomial test p < 0.05). Number of differentially methylated genes in each quadrant is indicated in blue and red, for all genes and differentially expressed genes, respectively. (B) Gene ontology and Reactome enrichment of genes with a negative correlation of gene expression and methylation. Lengths of bars represent negative ln-transformed, adjusted pvalues using Fisher’s exact test. Cells indicate log2-foldchanges (log2FC) between DR mice and control group per gene. (C,D) Venn diagrams depicting the overlap of genes being transcriptionally (C) down- or (D) up-regulated in DR or Ames dwarf mice, respectively. Both down- and up-regulated genes showed a significant overlap between both longevity models (p-values; *** p<0.001, ** p<0.01, * p<0.05, Fisher’s exact test). (E) Venn diagram depicting the overlap of genes being transcriptionally down-regulated and hypermethylated in DR and Ames dwarf mice. (F) Differential methylation landscape of the Fgfr3 gene locus in DR, Rapamycin treated and Ames dwarf mice. Bins are represented as bars with color scale and height indicating methylation differences. Arrows indicate gene orientation; merged mRNA structure is depicted below. (G) Fgfr3 mRNA expression by RNA-sequencing in control and Ames Dwarf mice at young and old age, respectively (n = 4 vs 2 and 4 vs 4). (H) Fgfr3 mRNA expression by RNA-sequencing in control and DR mice at young and old age, respectively (n = 3 vs 3 and 3 vs 3). (TIF) [file pgen.1007766.s012.tif]

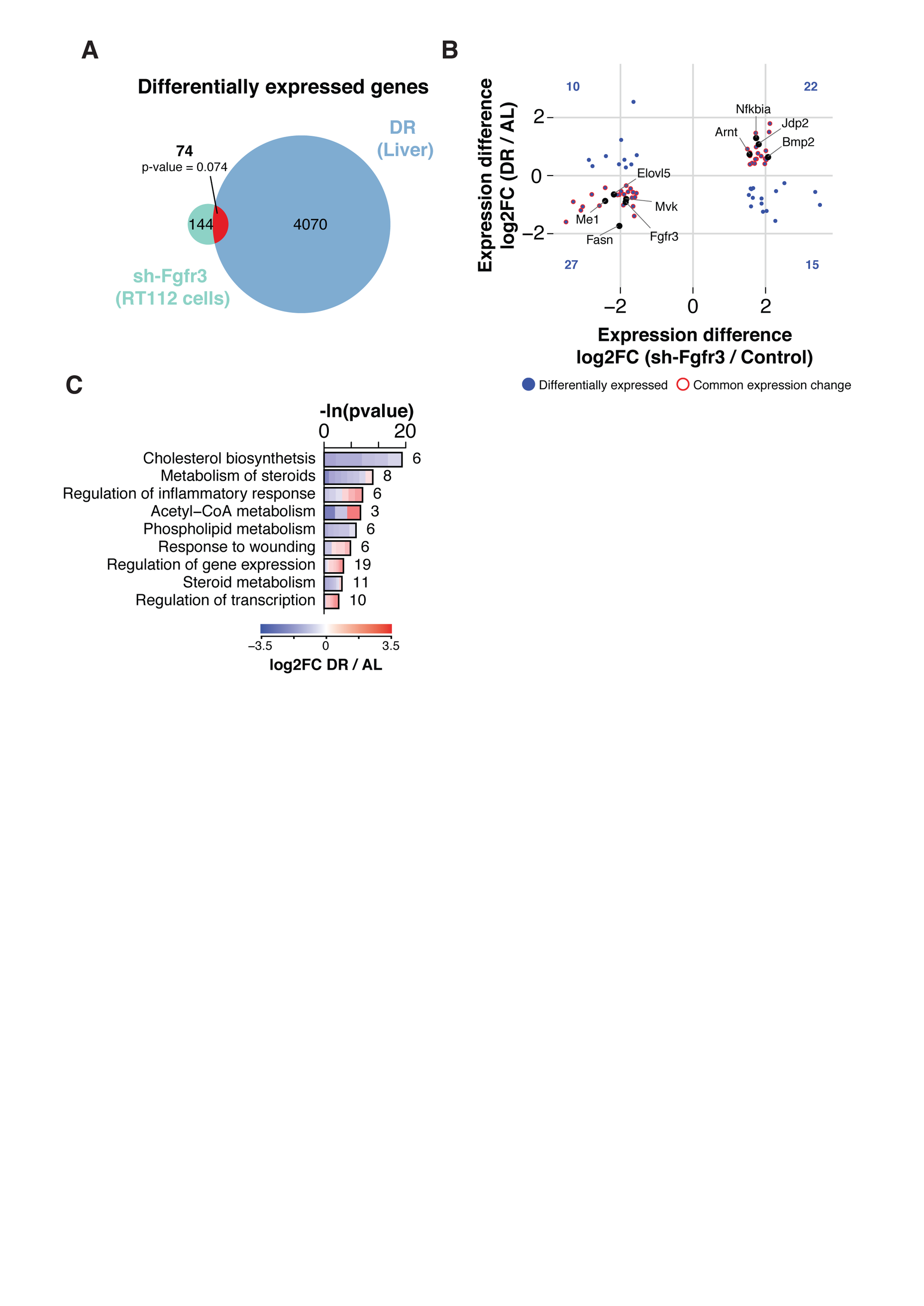

Supplement: S13 Fig — (A) Venn diagram depicting the overlap of genes differentially expressed in livers of DR treated mice and RT112 cells transfected with short-hairpin RNA targeting Fgfr3 (p-values; *** p<0.001, ** p<0.01, * p<0.05, Fisher’s exact test). (B) Scatterplot of expression differences in livers of DR treated mice versus expression differences in RT112 cells with induced Fgfr3 knockdown. Number of differentially expressed genes in each quadrant is indicated in blue with common expression changes highlighted in red. Both transcriptomes showed significantly common expression signatures (p < 0.01, Fisher’s exact test). (C) Gene ontology and Reactome enrichment of genes being commonly regulated in DR treated mice and RT112 cells with induced Fgfr3 knockdown. Lengths of bars represent negative ln-transformed, adjusted pvalues using Fisher’s exact test. Cells indicate log2-foldchanges (log2FC) between DR mice and control group per gene. (TIF) [file pgen.1007766.s013.tif]
